# Pom1 gradient buffering through intermolecular auto-phosphorylation

**DOI:** 10.15252/msb.20145996

**Published:** 2015-07-06

**Authors:** Micha Hersch, Olivier Hachet, Sascha Dalessi, Pranav Ullal, Payal Bhatia, Sven Bergmann, Sophie G Martin

**Affiliations:** 1 Department of Medical Genetics, University of LausanneLausanne, Switzerland; 2 Swiss Institute of BioinformaticsLausanne, Switzerland; 3 Department of Fundamental Microbiology, University of LausanneLausanne, Switzerland

**Keywords:** auto-catalysis, cell cycle control, fission yeast *Schizosaccharomyces pombe*, gradient formation, robustness

## Abstract

Concentration gradients provide spatial information for tissue patterning and cell organization, and their robustness under natural fluctuations is an evolutionary advantage. In rod-shaped *Schizosaccharomyces pombe* cells, the DYRK-family kinase Pom1 gradients control cell division timing and placement. Upon dephosphorylation by a Tea4-phosphatase complex, Pom1 associates with the plasma membrane at cell poles, where it diffuses and detaches upon auto-phosphorylation. Here, we demonstrate that Pom1 auto-phosphorylates intermolecularly, both *in vitro* and *in vivo*, which confers robustness to the gradient. Quantitative imaging reveals this robustness through two system’s properties: The Pom1 gradient amplitude is inversely correlated with its decay length and is buffered against fluctuations in Tea4 levels. A theoretical model of Pom1 gradient formation through intermolecular auto-phosphorylation predicts both properties qualitatively and quantitatively. This provides a telling example where gradient robustness through super-linear decay, a principle hypothesized a decade ago, is achieved through autocatalysis. Concentration-dependent autocatalysis may be a widely used simple feedback to buffer biological activities.

## Introduction

Protein concentration gradients provide spatial information for cellular and developmental processes. Classical morphogen gradients translate cell position into distinct cell fate to pattern a developing organism (Rogers & Schier, 2011). They are often able to do so robustly despite fluctuations in morphogen production, though the underlying mechanisms are debated and subject of intense research (Barkai & Shilo, 2009; de Lachapelle & Bergmann, 2010; Howard, 2012; Saunders *et al*, 2012; Cheung *et al*, 2014). Gradients also occur at much smaller scales within cells, where they impart spatial cellular order, for instance in organizing the mitotic spindle or controlling cell division (Lutkenhaus, 2007; Fuller, 2010). In rod-shaped fission yeast *Schizosaccharomyces pombe* cells, the DYRK-family kinase Pom1 forms membrane-associated concentration gradients from cellular extremities (Padte *et al*, 2006; Hachet *et al*, 2011). This kinase controls the timing and positioning of cell division by inhibiting the activity and restricting the localization of its substrate Cdr2 at the cell equator (Martin & Berthelot-Grosjean, 2009; Moseley *et al*, 2009; Bhatia *et al*, 2013; Deng *et al*, 2014; Rincon *et al*, 2014). Pom1 reversibly binds the plasma membrane in a manner that depends on its degree of phosphorylation (Hachet *et al*, 2011). Initiation of Pom1 gradients relies on dephosphorylation of Pom1 by a type I phosphatase complex, whose regulatory subunit Tea4 is actively transported to cell extremities by microtubules (Martin *et al*, 2005; Tatebe *et al*, 2005; Alvarez-Tabares *et al*, 2007; Hachet *et al*, 2011). At the plasma membrane, Pom1 diffuses laterally and auto-phosphorylates, which promotes detachment from the plasma membrane and thus forms a gradient that decays toward the cell middle (Hachet *et al*, 2011).

## Results

**Figure 1:**
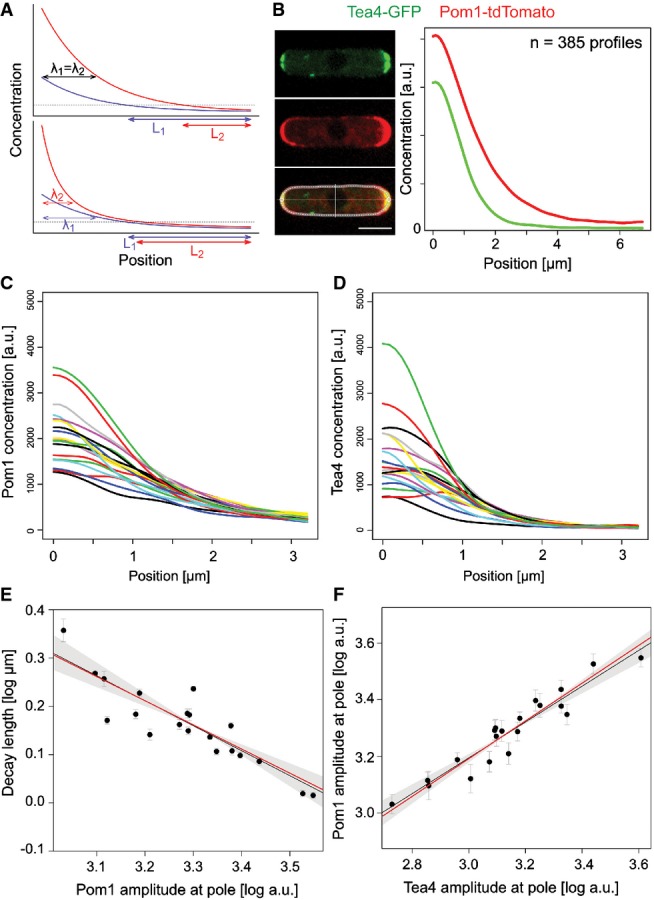
Quantitative analysis of Pom1 gradient reveals two system’s properties buffering against high variability of Tea4 at cell poles The standard diffusive gradient (top) is exponential, and its decay length λ (the distance at which the concentration is reduced to some fraction of its amplitude) is independent from the amplitude. Variations in amplitudes are therefore not buffered and translate into large variations in positional information L. By contrast, a gradient that reduces its decay length at high amplitudes (bottom) buffers variations in amplitude and conveys more robust positional information (L1 ≈ L2).

The Pom1 gradient has been shown to be highly robust against fluctuations in amplitude at the pole, with higher Pom1 peak levels usually coinciding with a smaller gradient decay length, indicative of a mechanism buffering Pom1 levels at cell sides (Saunders *et al*, 2012; Fig1A). To describe the shape of Pom1 gradients quantitatively, we measured Pom1-tdTomato and Tea4-GFP distributions in 97 cells (388 distinct profiles) using the Cellophane ImageJ plugin (Bhatia *et al*, 2013; Fig1B). GFP fluorescence measurements have been previously shown to be linearly related to protein concentrations (Wu & Pollard, 2005). Across cells, we observed a variation of cortical and total Pom1 amounts, as well as cytoplasmic Pom1 concentration slightly above twofold, as would be expected from cells that repeatedly half and then double their volume along the cell cycle, hinting at a possible control of Pom1 production and degradation. Pom1 levels at cell poles display a somewhat higher variability with up to several fold differences in amplitude across cells (Saunders *et al*, 2012) (Supplementary Fig S1D) and two- to fourfold differences within cells (Supplementary Fig S1B).

Pom1-tdTomato (red) and Tea4-GFP (green) profiles were quantified along the cortex of the same *Schizosaccharomyces pombe* cells. Curves are averages from 385 individual profiles.

Smoothed Pom1 gradients averaged in batches of 5% (*n* = 19 or 20) from lowest to highest Tea4 amplitude. Amplitudes at the pole show a high variability.

Corresponding smoothed and averaged Tea4 profiles. The variability at the pole is even higher than for Pom1.

Pom1 gradient decay length decreases with the amplitude at the pole. Each dot corresponds to one average profile as in (C). The *trans*-phosphorylation model predicts a slope of −1/2 in log–log space (red line), close to the observed linear regression of −0.52 (black line) and well within the two SE confidence interval (shaded area). Each data point is weighted by its inverse variance of the mean in the linear regression.

**Figure 2:**
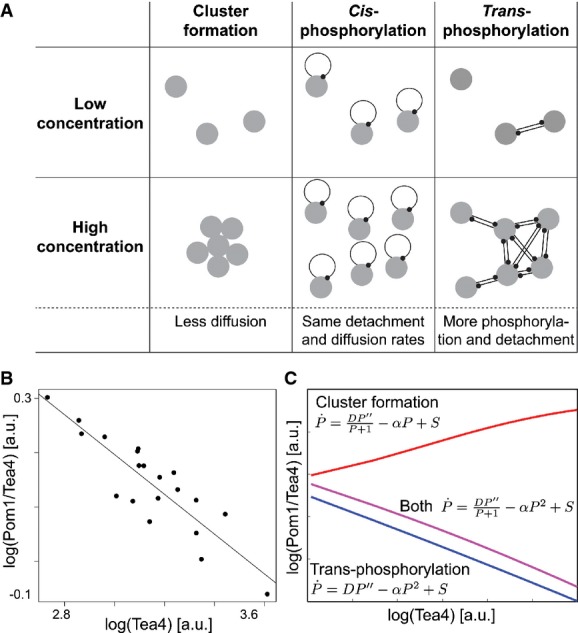
A model of Pom1 intermolecular auto-phosphorylation predicts Pom1 gradient’s system properties Possible hypotheses for a buffering mechanism. In the cluster-based model, high local concentration leads to the formation of slow-diffusing clusters, thus reducing the decay length at cell poles with high amplitude (Saunders *et al*, 2012). A *cis*-phosphorylation model provides no buffering. By contrast, in a *trans*-phosphorylation model, phosphorylation (and thus detachment) increases with local Pom1 concentration, providing a buffering mechanism reducing decay length at cell poles with high amplitude.

Pom1 amplitude increases with Tea4 concentration at the pole. Linear regression (black line) in the log–log scale shows a slope smaller than one indicative of a sub-linear relationship between Pom1 and Tea4 at the pole (see also Fig2B) and thus buffering of Tea4 fluctuations. Each dot corresponds to one average Tea4 and corresponding Pom1 profiles as in (C, D). The *trans*-phosphorylation model predicts the slope to be 2/3 (red line), close to the observed 0.63 (black line), and within the two SE confidence interval (shaded area). Each data point is weighted by its inverse variance of the mean in the linear regression. Data information: Error bars represent the s.e.m., and all logarithms are in base 10.

Similar to previous results, we show that Pom1 gradient shape adapts to this variability, as the Pom1 gradient decay length shows a strong negative correlation with the Pom1 amplitude (Saunders *et al*, 2012) (Fig1E). Variation in total Pom1 amounts across cells (Supplementary Fig S1A), covariation of cortical and cytosolic amounts of Pom1 across cells (Supplementary Fig S1C), and a strong correlation between these cortical amounts and the Pom1 amplitude at the cell pole (Supplementary Fig S1F) indicate that this negative correlation between decay length and amplitude cannot be explained by a mechanism that would keep the total amount of cortical Pom1 (and thus the area under the profile) constant across cells. Moreover, the coefficient of variation of the Pom1 concentration decreases away from the cell pole (Supplementary Fig S2), suggesting that this negative correlation is the result of a buffering mechanism on the decay length rather than a tight regulation of total cortical Pom1.

Tea4 levels at cell poles showed even higher relative variability than Pom1 (Bartlett test, *P*-value = 0.039) and were highly correlated with Pom1 levels (Fig1D, F). However, the relationship between Pom1 and Tea4 levels is not proportional, as the ratio between Pom1 and Tea4 amplitudes was strongly negatively correlated with that of Tea4 (Supplementary Figs S2B and S5A). This observation indicates that Pom1 amplitude is buffered against variations in Tea4 levels, which are probably caused by the discontinuous delivery of the phosphatase regulatory subunit Tea4 to cell poles upon each microtubule contact (Martin *et al*, 2005; Tatebe *et al*, 2005).

Previous work proposed that the concentration-dependent formation of slow-diffusing Pom1 clusters may underlie Pom1 gradient shape buffering, where high Pom1 concentration would form larger, slower-diffusing clusters, leading to a steep gradient (Saunders *et al*, 2012). This hypothesis implies that a high Pom1 influx to the cell pole would increase its local accumulation through to a “traffic jam” phenomenon because the more Pom1 is brought to the cell pole, the less it can diffuse away. This prediction is contradictory to the observed reduced variability of Pom1 compared to Tea4 concentration at the pole and negative correlation between Pom1/Tea4 and Tea4 (see Fig2B). Indeed, a simplified “cluster-based” model, in which Pom1 diffusion decreases with its local concentration, reproduced the observed negative correlation between Pom1 gradient amplitude and decay length (Supplementary Fig S3), but predicted a positive correlation between the Pom1/Tea4 ratio and Tea4, incompatible with our experimental data (Fig2C). Similarly, the detailed two-component clustering model described by Saunders *et al* (2012) also predicted such positive correlation (Supplementary Fig S4). Together, these data confirm that adapting diffusion is not sufficient for explaining the observed buffering of Pom1 against Tea4 levels.

Inverse correlation between Pom1/Tea4 and Tea4 observed experimentally following from the sub-linear relationship between Pom1 and Tea4 (see Fig1F, same data). Line shows linear regression (*P*-value < 10^−7^).

Relationship between Pom1/Tea4 and Tea4 predicted by the simple cluster-based and *trans*-phosphorylation models. The simple cluster-based model simply reflects the hypothesis that diffusion decreases with Pom1 concentration P. It illustrates that this hypothesis cannot alone explain the decreasing relationship between Pom1/Tea4 and Tea4 (red line). By contrast, this relationship is well accounted for qualitatively and quantitatively by the *trans*-phosphorylation model (blue line; see also 1F). Combining both Pom1 clustering and *trans*-phosphorylation can also account for the decreasing relationship between Pom1/Tea4 and Tea4 (purple line). Data information: All logarithms are in base 10.

We considered the distinct (but not mutually exclusive) hypothesis that concentration-dependent modulation of Pom1 auto-phosphorylation, and thus detachment rate, may explain both Pom1 gradient properties, that is, the negative correlation between the Pom1 gradient amplitude and decay length, and the negative correlation between Pom1/Tea4 ratio and Tea4 levels. Pom1 auto-phosphorylation may occur intramolecularly (in *cis*) or intermolecularly between two distinct Pom1 molecules (in *trans*). In the *cis* configuration, Pom1 auto-phosphorylation is independent of Pom1 local concentration. By contrast, if two Pom1 molecules phosphorylate in *trans*, then phosphorylation is directly linked to the local Pom1 concentration (Fig2A). Intuitively, Pom1 *trans*-phosphorylation could explain a negative correlation between Pom1 gradient amplitude and decay length, with higher local Pom1 levels causing higher *trans*-phosphorylation and thus detachment rate, leading to a steeper gradient.

To formally explore this hypothesis, we considered a mathematical model of Pom1 gradient formation through intermolecular phosphorylation. Previous experimental data showed that Pom1 auto-phosphorylates on multiple sites, at least 6 of which control its membrane affinity (Hachet *et al*, 2011). Taking into account the various phosphorylation states of Pom1 as distinct Pom1 species gives rise to the detailed model described in Box 1. Analytical developments combined with numerical simulations presented in the Supplementary Text S1 show that if the Pom1 detachment rate increases at least linearly with its number of phosphorylated residues, this model is well approximated by a much simpler model described by 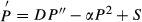, where *P* is the total cortical Pom1 concentration, *D* the diffusion constant, and α an effective detachment rate. *S* is the influx of Pom1 at the cell tip and can be experimentally quantified (up to a multiplicative constant) by the Tea4-GFP signal. We note that the concentration of cytosolic Pom1 (which represents a significant fraction of all Pom1 in the cell; Supplementary Fig S1E) does not appear to strongly influence S because we observe no correlation between the amplitude of Pom1 at the pole and Pom1 concentration in the cytoplasm (Supplementary Fig S1B and D).

**Box 1: Mathematical results**

If *P*_*i*_ (*x*, *t*) is the cortical concentration of Pom1 phosphorylated *i* times, the total Pom1 concentration is given by 
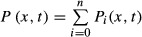
. Pom1 is brought to the tip by the Dis2-Tea4 complex at a rate *S*(*x*), diffuses along the cortex with a coefficient *D*, and phosphorylates intermolecularly with a rate *β*. Phosphorylated Pom1 molecules detach with increasing rate *κ*_*i*_ < *κ*_*i*+1_, resulting in the following dynamics: 

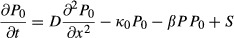


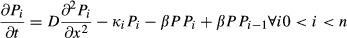


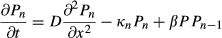

Summing those equations yields

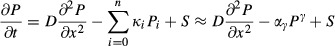
 where *α*_*γ*_ is the effective detachment rate and 1.5 < *γ* ≤ 2. The above approximation is validated by numerical simulations. Theoretical considerations (see Supplementary Text S1) along with experimental data indicate that *γ* ≅ 2, resulting in the following model: 

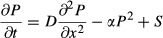

The solution at steady state is given by 

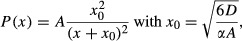
 where the length scale *x*_0_ is proportional to the decay length of the profile, *λ*, and *A* is the gradient amplitude at the pole and can be computed as 

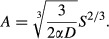

The total amount of Pom1 *P*_tot_ in the gradient is given by 
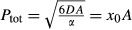
.

Upon a *k*-fold increase in the Pom1 attachment rate *S*, a position given by a threshold concentration of Pom1 is shifted by 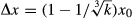, which is always less than *x*_0_. See Supplementary Text S1 for more details.

This simple model can be solved analytically and leads to a gradient profile that decays as a power of the distance from the source (in contrast to an exponential decay in case of cis-phosphorylation corresponding to a linear decay term) (Eldar *et al*, 2003; Wartlick *et al*, 2009). The solution predicts that the Pom1 gradient decay length λ decreases with the inverse square root of the amplitude at the origin, amounting to a power law with a coefficient of −1/2 between the Pom1 amplitude and the decay length (this coefficient *p* is the slope of [Pom1] vs. λ in log–log space). This prediction matches well our experimental measurements (*p *= −0.52 ± 0.06 (SE); Fig1E). Equivalently, the model predicts a 1/2 power law between the Pom1 amplitude and the overall cortical Pom1, closed to the observed value of 0.46 (Supplementary Fig S1F). Furthermore, this simple *trans*-phosphorylation model also predicts a power law relationship with *p *= 2/3 between the Pom1 amplitude at the pole and Tea4 (which is proportional to *S* in Box 1), consistent with the experimentally observed power law (*p *= 0.63 ± 0.05 (SE), Figs1F and 2B–C and Supplementary Fig S5A and B). Finally, we show that individual profiles can be fit equally well with an exponential or a power law (Supplementary Fig S6). Thus, our model of trans-phosphorylation explains the observed system properties of Pom1 gradient and accurately predicts quantitative aspects of these properties.

Using a combination of the simplified “cluster-based” model (described above and in Fig2C and Supplementary Figs S3 and S5) and our simple *trans*-phosphorylation model, we further show that the *trans*-phosphorylation model can have a dominant effect over the clustering model to explain the negative correlation between Pom1/Tea4 and Tea4, indicating that both models could coexist (Fig2C).

**Figure 3:**
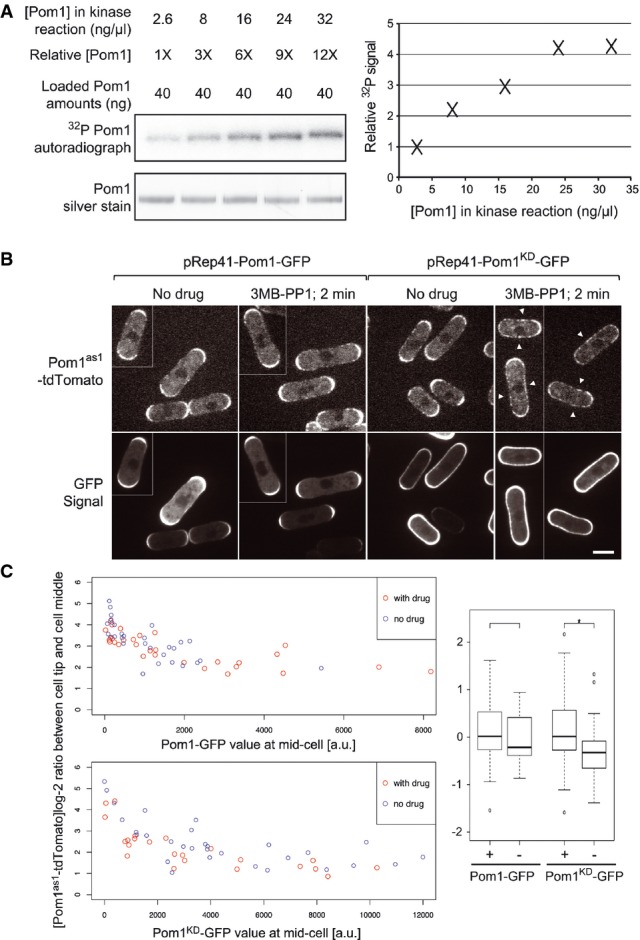
Pom1 auto-phosphorylates intermolecularly *in vitro* and *in vivo* Pom1 *in vitro* kinase assays with [ϒ-^32^P] ATP at five distinct Pom1 concentrations, ranging from 2.6 to 32 ng/μl (1× to 12×). Loading of equivalent Pom1 amounts (40 ng; see silver staining control) reveals higher incorporation of ^32^P upon higher Pom1 concentration, indicating an intermolecular reaction. Quantification is shown on the graph.

To directly address whether Pom1 auto-phosphorylation occurs in *trans in vitro*, we performed kinase assays with γ^32^P-labeled ATP at different Pom1 concentrations (Figs3A and Supplementary Fig S7). Phosphorylation rates are predicted to increase with Pom1 concentration only if auto-phosphorylation is intermolecular. By comparing equivalent amounts of Pom1 from reactions at different concentrations, we observed that ^32^P incorporation increases with Pom1 concentration. Furthermore, we found the Pom1 can phosphorylate an inactive Pom1^KD^ allele (lacking the first 305 aa so it can be distinguished from wt Pom1 on the gel) *in vitro* (Supplementary Fig S7D). Thus, Pom1 auto-phosphorylates in *trans in vitro*.

Pom1^as1^-tdTomato in cells co-expressing Pom1-GFP (left) or Pom1^KD^-GFP (right) before and 1–2 min after the addition of 3MB-PP1, as indicated. Before drug addition, Pom1 is strongly enriched at cell poles. After drug addition, Pom1^as1^-tdTomato is rapidly delocalized around the entire cell periphery (arrowheads) in cells co-expressing inactive Pom1^KD^-GFP, but not in cells co-expressing active Pom1-GFP, suggesting intermolecular phosphorylation (Hachet *et al*, 2011). Medial confocal planes are shown. Scale bar is 5 μm.

Quantification of data shown in panel B for a larger number of cells. The graphs on the left show the log-2 ratio of Pom1^as1^-tdTomato fluorescence levels at cell tip and cell middle relative to the medial Pom1-GFP (top) or Pom1^KD^-GFP (bottom) signal. Note that the Pom1^as1^-tdTomato tip/middle log-2 ratios are lowered by 3MB-PP1 addition (red dots) relative to the non-treated samples (blue dots) in the cells co-expressing Pom1^KD^-GFP, but not in the cells co-expressing Pom1-GFP. A boxplot (right) of the residuals after correction of the effect of the GFP signal illustrates the significant effect of the drug treatment on Pom1^as1^-tdTomato localization in cells co-expressing Pom1^KD^-GFP (*n = 30 + 23, one-sided *t*-test, *P*-value =0.012) but not in cells expressing Pom1-GFP (*n *= 30 + 30, two-sided *t*-test, *P*-value = 0.39). + and − indicate the presence or absence of 3MB-PP1.

We used Pom1 localization as readout of Pom1 phosphorylation state to test whether Pom1 *trans*-phosphorylates *in vivo*. Indeed, inactive or non-phosphorylatable Pom1 alleles bind the plasma membrane more strongly and decorate the entire cell periphery because they do not rely on local dephosphorylation by the Tea4-PP1 complex for membrane binding (Hachet *et al*, 2011). We used a Pom1^as1^ allele, which is initially functional and localized as wild-type Pom1 at cell poles, but can be acutely inhibited by the addition of an ATP analogue 3MB-PP1 (Padte *et al*, 2006; Bhatia *et al*, 2013). We previously showed that Pom1^as1^-tdTomato rapidly delocalizes over a large portion of the cell cortex within 3 min of 3MB-PP1 addition (Hachet *et al*, 2011). We reasoned that in case of intermolecular phosphorylation, co-expression of wild-type Pom1 may slow down the kinetics of Pom1^as1^ delocalization. Indeed, co-expression of wild-type Pom1-GFP maintained Pom1^as1^-tdTomato cell tip localization within 1–2 min after 3MB-PP1 addition (Fig3B). By contrast, co-expression of Pom1^KD^-GFP did not prevent Pom1^as1^-tdTomato delocalization (Fig3B). High expression of Pom1^KD^-GFP also appeared to have a drug-independent effect on Pom1^as1^-tdTomato localization, possibly through protein–protein interaction. We thus used a linear model with the GFP signal at mid-cell as a covariate to assess the effect of the drug treatment on Pom1^as1^-tdTomato localization quantified as the log-ratio of its concentration at the cell pole and the cell middle (Fig3C). This showed that the drug treatment had a small yet significant effect on Pom1^as1^-tdTomato localization in cells co-expressing Pom1^KD^-GFP (*n* = 30 + 23, one-sided, *P*-value =0.012) but not in cells expressing Pom1-GFP (*n* = 30 + 30, two-sided, *P*-value = 0.39). These results are in agreement with the idea that Pom1-GFP phosphorylates Pom1^as1^-tdTomato to delay its delocalization, providing *in vivo* evidence for Pom1 *trans*-phosphorylation. We note that our *in vivo* data cannot exclude that Pom1 may also auto-phosphorylate in *cis*. Sustained drug treatment eventually led to Pom1^as1^-tdTomato redistribution over the entire cell periphery even in the presence of Pom1-GFP as observed in the case of Pom1^KD^ (Bähler & Nurse, 2001; Hachet *et al*, 2011), likely because inactive Pom1^as1^ or Pom1^KD^ on cell sides overlaps only very poorly with wild-type Pom1 at cell poles (data not shown). In conclusion, *in vitro* and *in vivo* data are consistent with the idea that Pom1 auto-phosphorylates intermolecularly.

## Discussion

*In vitro* and *in vivo* evidence indicates that Pom1 *trans*-phosphorylates *in vivo*, and cell population analysis of Pom1 gradient shape exhibits the exact system properties expected from such a *trans*-phosphorylation-induced Pom1 detachment: a −1/2 power law between the gradient amplitude and decay length and a 2/3 power law between the Tea4 concentration and the Pom1 concentration at the pole. Such population-level properties are much more powerful for distinguishing between different models than the shape of individual gradient profiles (see Supplementary Fig S6). We conclude that the Pom1 gradient is buffered against fluctuation in attachment rate through intermolecular auto-phosphorylation. This, however, does not exclude that Pom1 also auto-phosphorylates intramolecularly. Similarly, although a previously proposed concentration-dependent effect on diffusion rate (Saunders *et al*, 2012) cannot explain both system’s properties, it may coexist with the concentration-dependent effect on Pom1 phosphorylation and membrane detachment proposed here, possibly even acting cooperatively by reducing the gradient length scale.

Conceptually, similar nonlinear self-regulatory mechanisms have been shown to buffer morphogen gradients against variations in developing embryos and tissues (Eldar *et al*, 2003; White *et al*, 2007). In these cases, the morphogen, by inducing cell signaling, indirectly promotes its own degradation in a concentration-dependent manner. By contrast, the concentration-dependent release of Pom1 from the membrane is directly induced by Pom1 action on itself. Indeed, by promoting Pom1 detachment from the membrane, the same biochemical reaction—Pom1 intermolecular auto-phosphorylation—underlies the decay of the gradient away from its site of membrane association at cell poles (Bähler & Nurse, 2001; Hachet *et al*, 2011) and provides a concentration-dependent negative feedback that buffers Pom1 gradients against variations. Beyond concentration gradients, analogous negative feedbacks may arise in many other kinase systems, including *trans*-auto-phosphorylation of the myosin IIIa, an unconventional myosin possessing a kinase domain, to down-regulate its own localization at the tips of filopodia (Quintero *et al*, 2010); *trans*-auto-phosphorylation of PLK4, a major kinase for centriole biogenesis, to promote its degradation and prevent excessive centriole number (Cunha-Ferreira *et al*, 2013); or *trans*-phosphorylation of Src to promote its inactivation (Osusky *et al*, 1995). Thus, intermolecular autocatalysis may represent a simple, built-in control mechanism to buffer biological activities.

## Supporting Information

**Supplementary Text S1**

**Review Process File**
