# Supplementary figures and images for: Pom1 gradient buffering through intermolecular auto-phosphorylation
Source: Mol Syst Biol. 2015 Jul 6;11(7):818. doi: 10.15252/msb.20145996 (PMC4547846; doi:10.15252/msb.20145996)

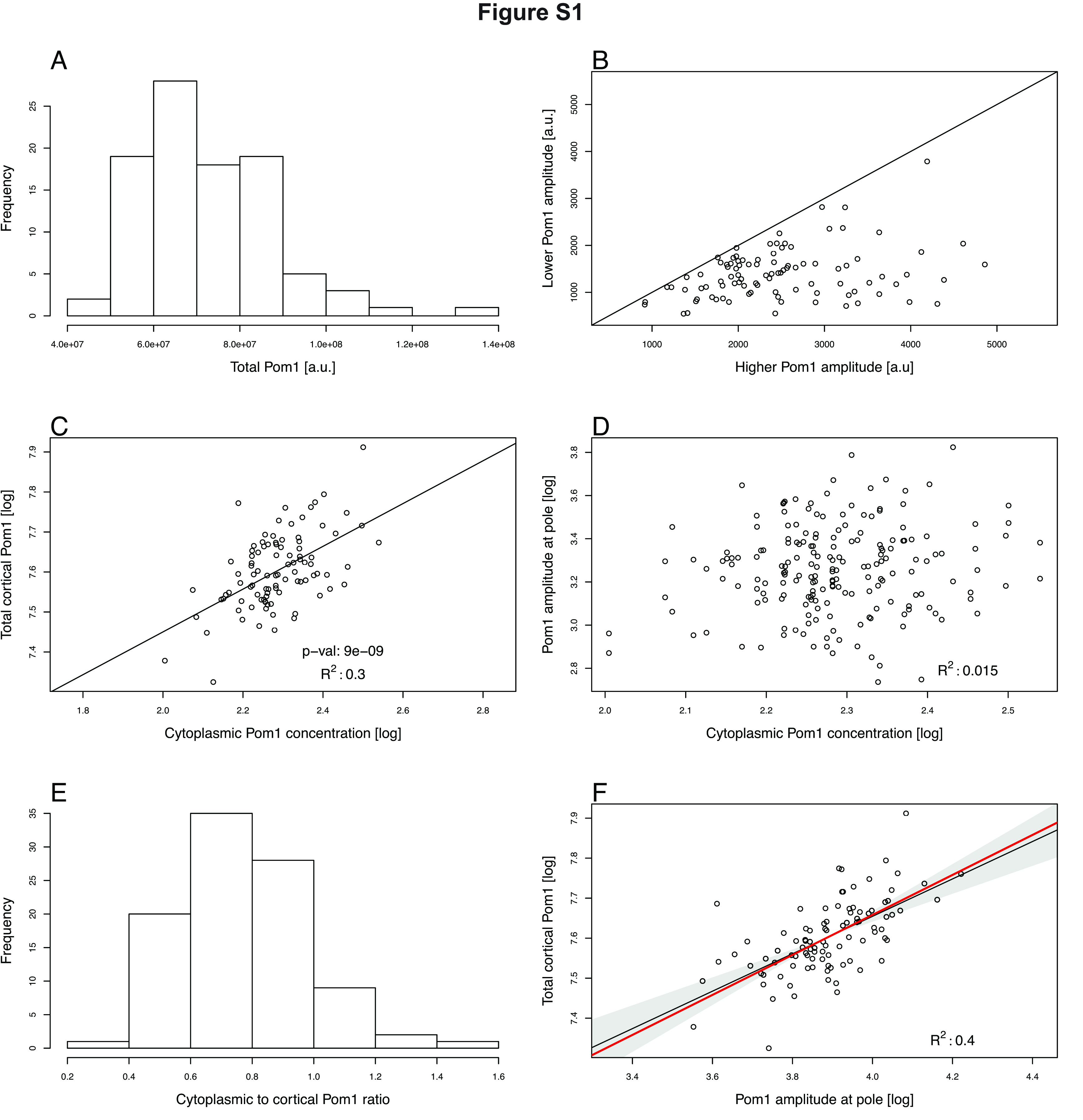

Supplement: Supplementary file 1 [file msb0011-0818-sd1.jpg]

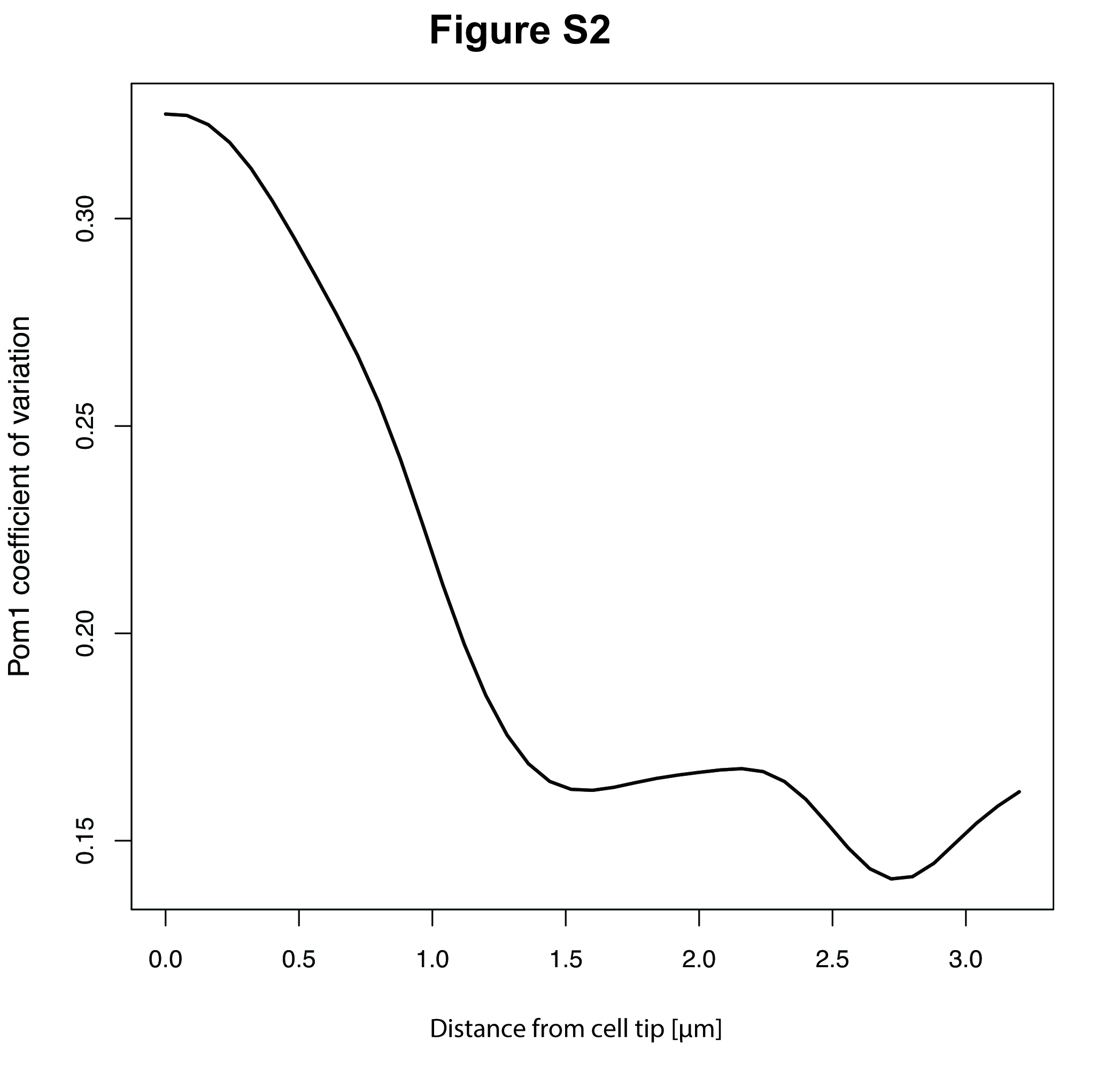

Supplement: Supplementary file 2 [file msb0011-0818-sd2.jpg]

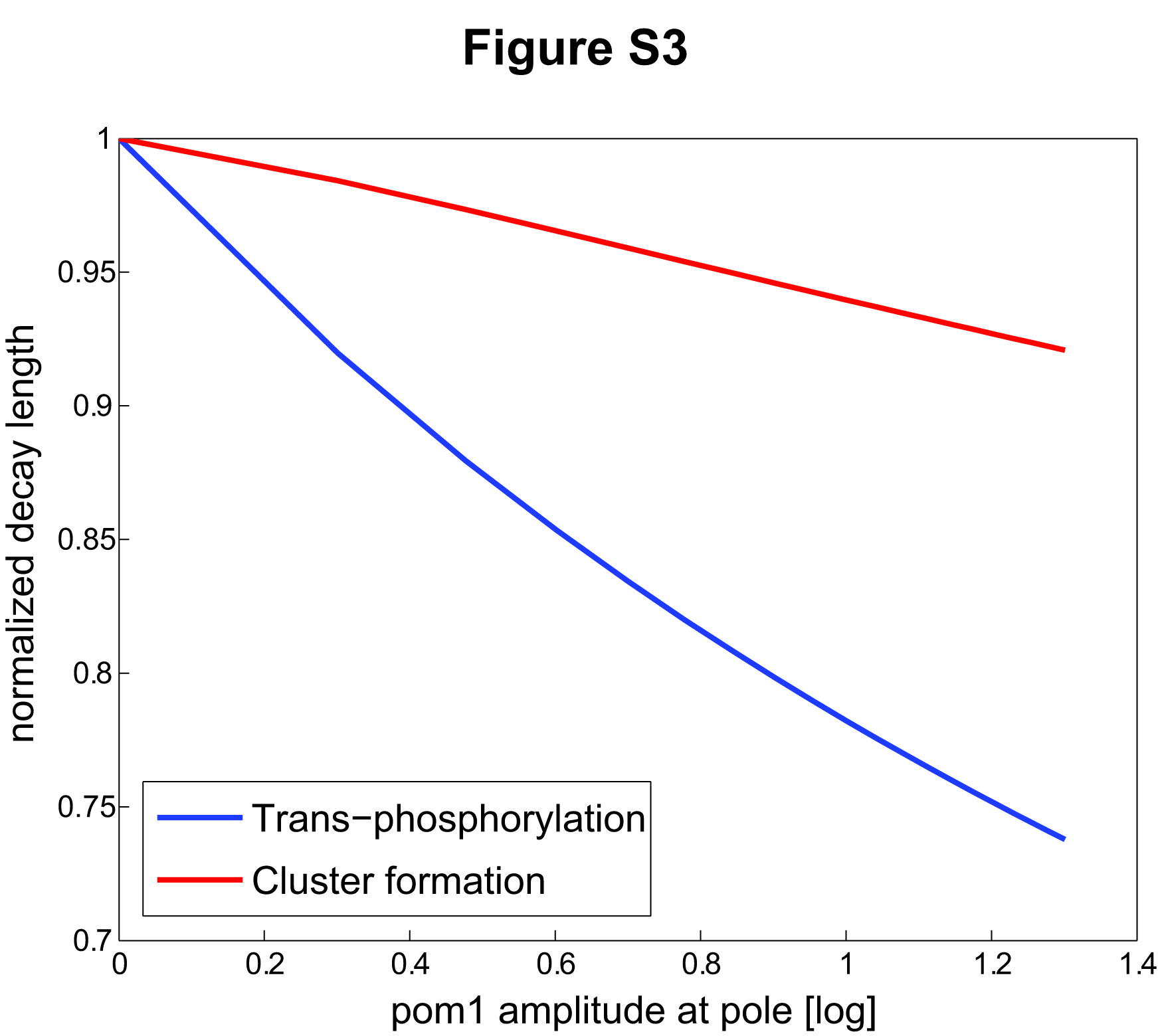

Supplement: Supplementary file 3 [file msb0011-0818-sd3.jpg]

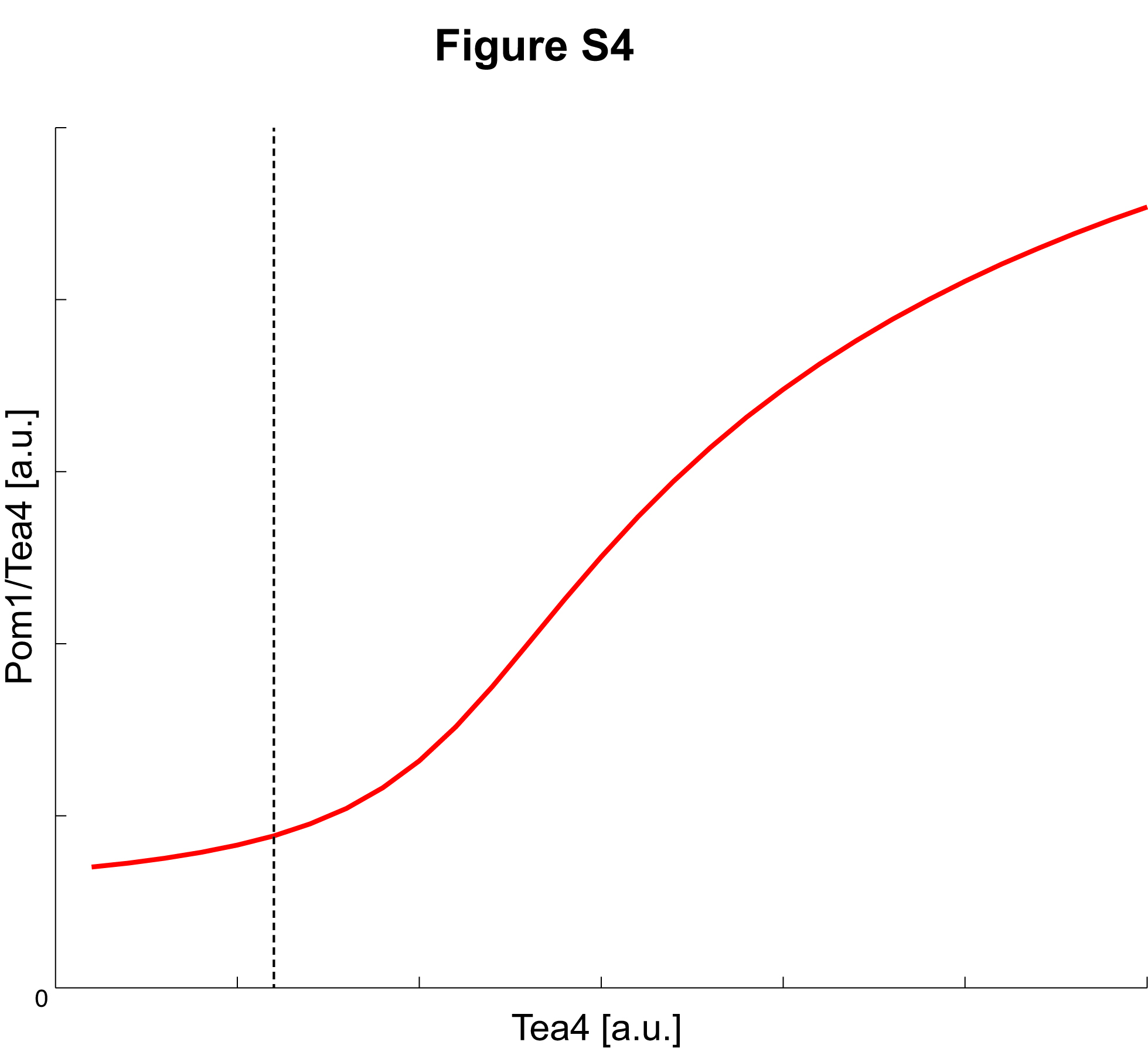

Supplement: Supplementary file 4 [file msb0011-0818-sd4.jpg]

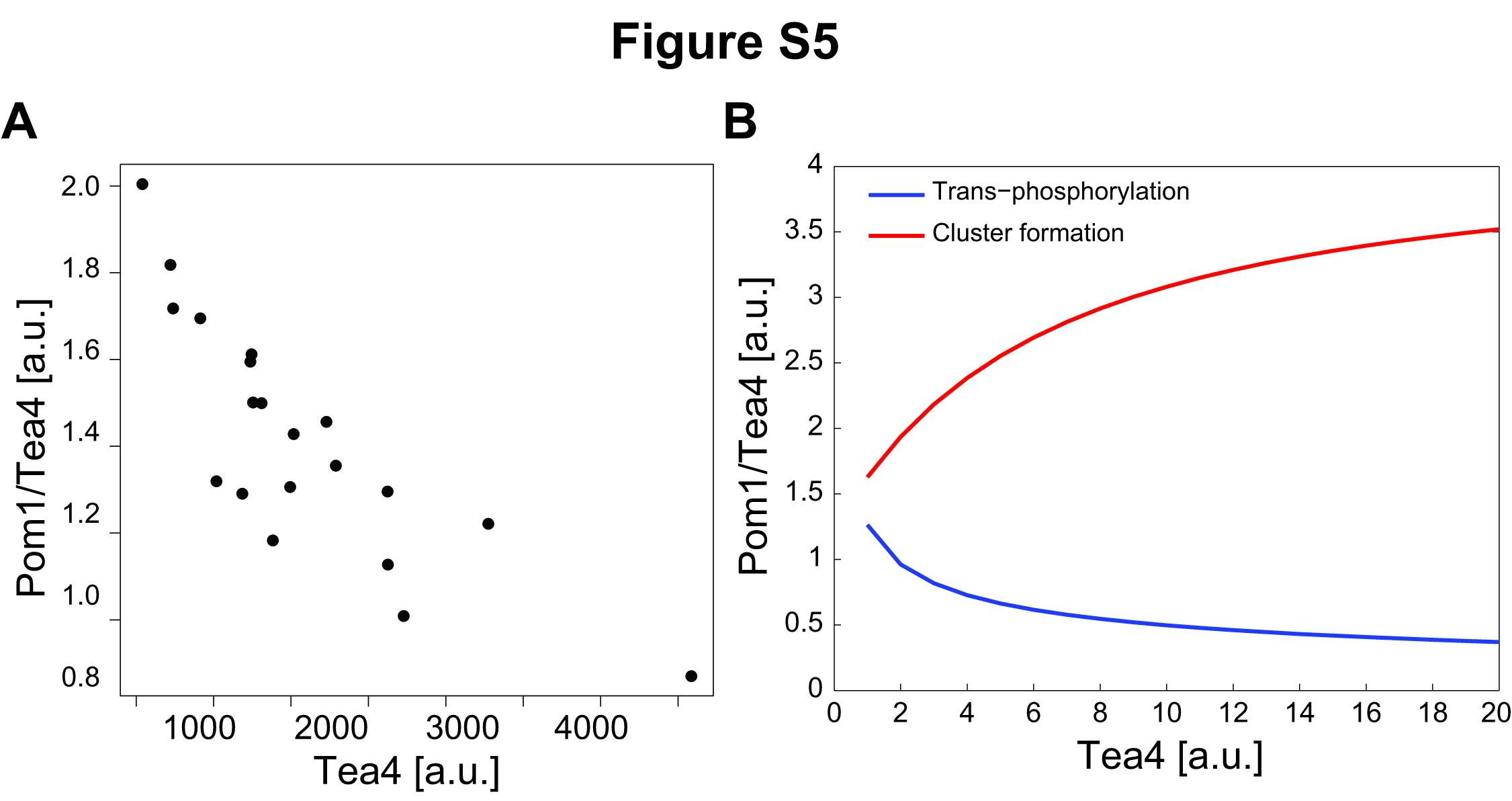

Supplement: Supplementary file 5 [file msb0011-0818-sd5.jpg]

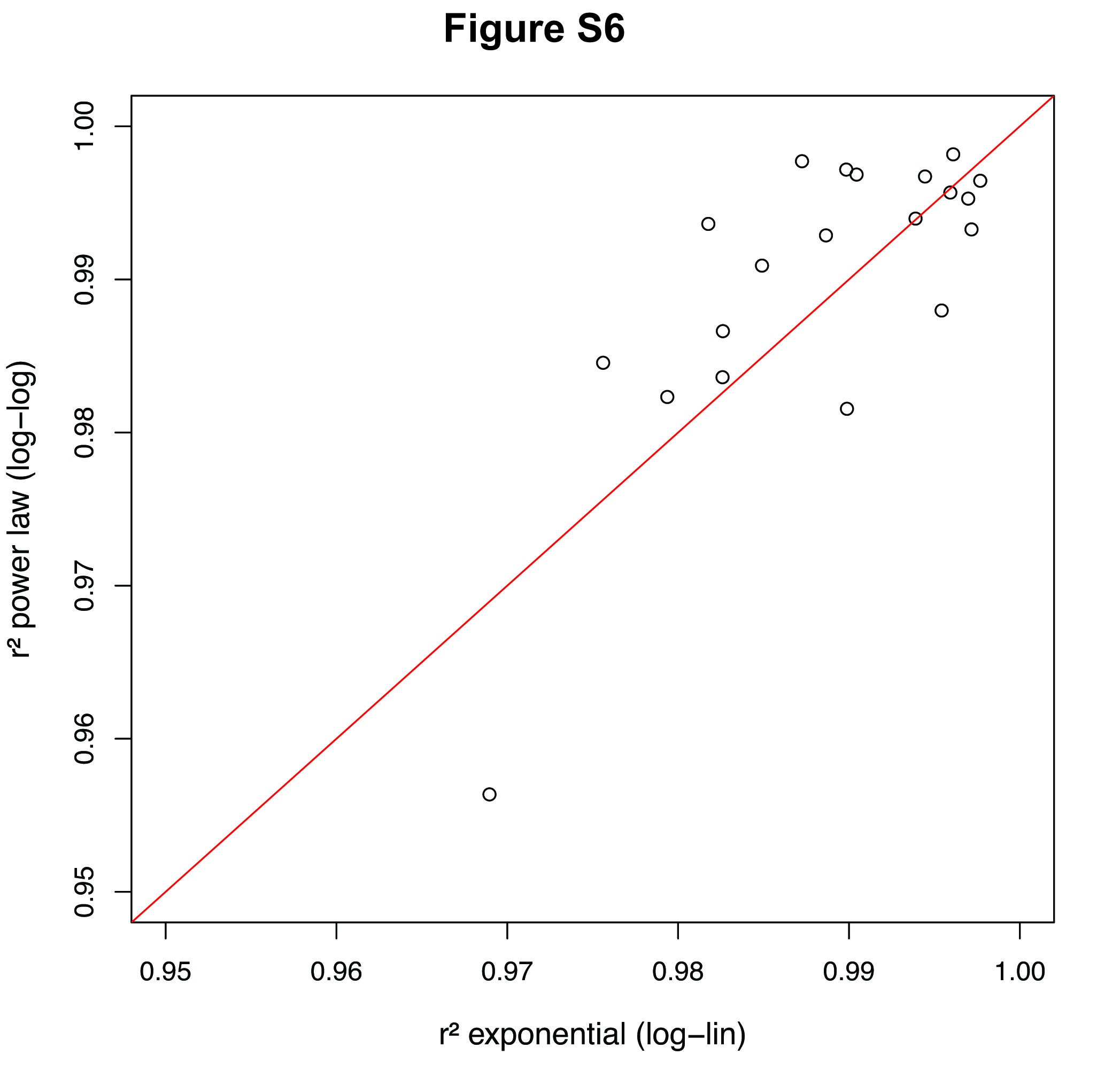

Supplement: Supplementary file 6 [file msb0011-0818-sd6.jpg]

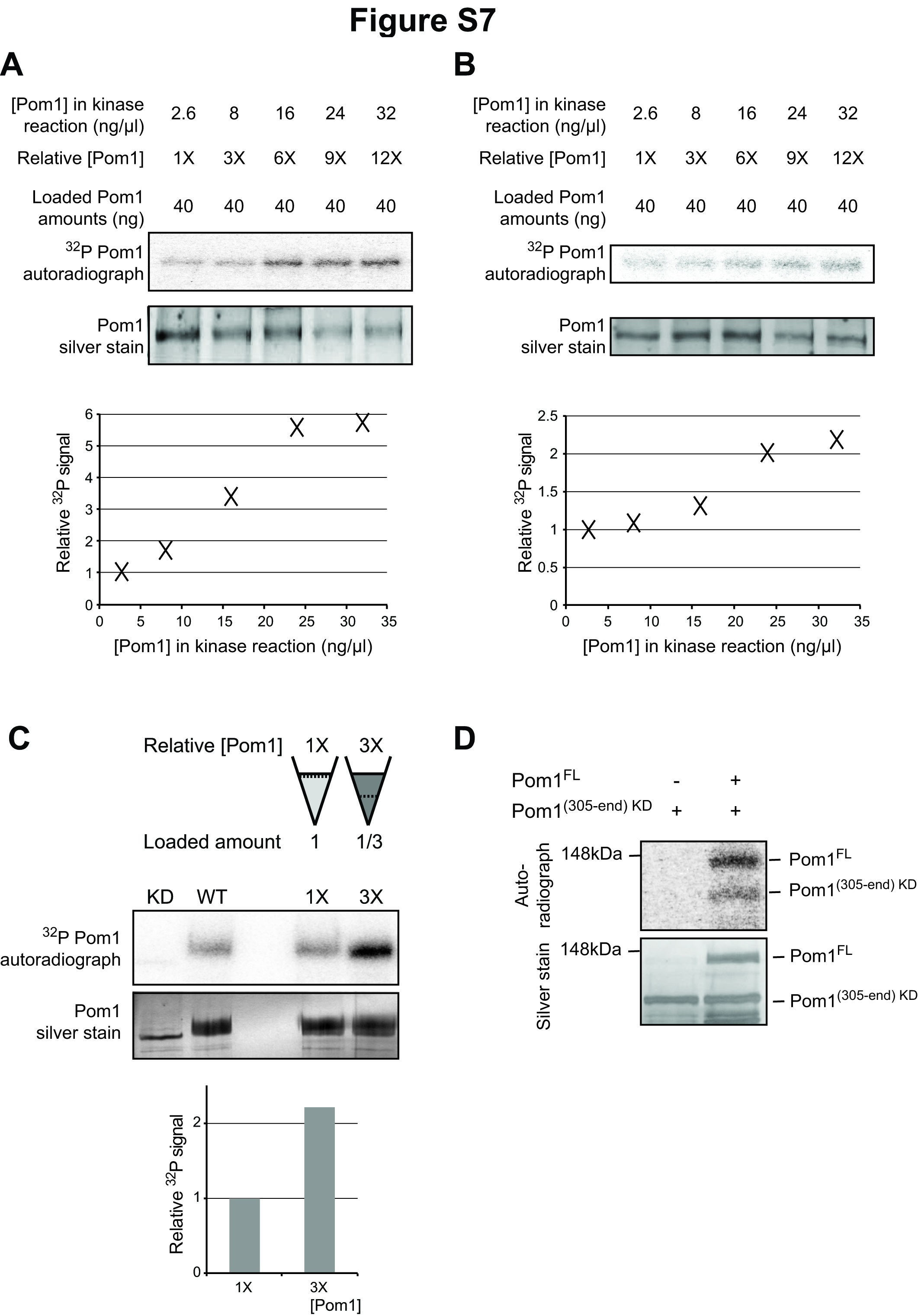

Supplement: Supplementary file 7 [file msb0011-0818-sd7.jpg]
