# Supplementary material for: Pom1 gradient buffering through intermolecular auto-phosphorylation
Source: Mol Syst Biol. 2015 Jul 6;11(7):818. doi: 10.15252/msb.20145996 (PMC4547846; doi:10.15252/msb.20145996)
Supplement: Supplementary file 8 [file msb0011-0818-sd8.docx]

**Supplementary Information**

**Supplementary Materials and methods**

Strain construction and culture

Standard *S. pombe* media and genetic manipulations were used throughout (Moreno et al, 1991). All strains used in the study were isogenic to wild-type 972 and are described in Table S1. Transformations were performed using the lithium acetate-DMSO method as described (Bähler et al, 1998). Standard molecular biology techniques were used to create all pREP41 plasmids. Cells were grown in exponential phase at 30°C in EMM supplemented with adenine and uracil. Thiamine was used at 0.1µM in EMM-AU to keep Pom1-GFP expression low. Cells were released from thiamine for 12-14hrs before imaging.

Microscopy, cell imaging and quantification

Imaging was done in EMM with the appropriate supplements at room temperature. Image acquisition was performed on a Perkin Elmer Leica DMI4000B inverted microscope equipped with an HCX PL APO × 100/1.46NA oil objective and a PerkinElmer Volocity Confocal system spinning disk microscope including a Yokagawa CSU22 real-time confocal scanning head, solid-state laser lines and a cooled 14-bit frame transfer EMCCD C9100-50 camera, as described (Bendezu et al, 2012).

For Pom1 and Tea4 signal quantifications with the Cellophane ImageJ plugin (http://www.unil.ch/cbg; (Bhatia et al, 2013), 5 pictures for each channel were acquired at maximal speed (1 s exposure time, binning 1). Quantification and processing was performed on an average projection of the 5 images, which provides some time-averaging smoothing of the distribution as described in (Bhatia et al, 2013). Briefly, the user manually marked cortex of the cell and the td-Tomato and GFP intensity signals are extracted over a width of three pixels. The positions on the profile of the cell poles are recorded and the background signal, estimated as the average signal intensity along the outskirt of the cell is subtracted. The cytoplasmic signal is similarly measured as the average signal along a closed curve that runs next to the membrane within the cell. We note that Pom1-GFP and Pom1-tdTomato distributions were previously shown to be indistinguishable, indicating that both fluorescent markers can be use to report on protein concentrations (Hachet et al, 2011; Pan et al, 2014). Because Pom1 gradient distribution is independent of cell length (Bhatia et al, 2013; Saunders et al, 2012), we did not consider cell length in this study, although it was also recorded.

Pom1^as1^-tdTomato time lapse imaging upon 3MB-PP1 addition was performed as mentioned above for cellophane quantifications. After spinning down the cells at 3000 rpm for 1 min, the drug was added and imaged immediately. Untreated control cells were imaged concomitantly during the same experiment.

Data analysis

For each of the 97 cells four gradient profiles were extracted. Upon visual inspection, 3 out of those 388 profiles were removed from the analysis. The profiles were smoothed using a Gaussian filter and aligned on the cell pole. They were then ordered according to the average Tea4 signal within 10 pixels (0.8 µm) from either side of the pole. The profiles were then binned by chunks of 5% and the average Tea4 and Pom1 profiles were computed for each of the 20 bins. The decay length of each of these average profiles was then computed as by performing a linear regression on the log value of the profile.

The comparison between the exponential and the power law fit was done by performing a linear regression either on the log-lin scale (exponential fit) or on the log-log scale (power law fit). To remove the effect of the flattening of the profile at the pole, the 10 first pixels (0.8 um) were not used for the fitting. The cytoplasmic concentration was obtained by removing the background signal from the cytoplasmic signal. The amount of Pom1 in the cytoplasm was computed by multiplying this value by the volume of the cell from which the volume of the Pom1-free nucleus was removed. The cell was approximated by capsule (a cylinder with two half spheres) and the nucleus by a sphere. Only the length of the cylinder was adapted from cell to cell, while the rest of the parameters were estimated from a subset of about 20 cells. The total amount of cortical Pom1 was computed by fitting the profiles on a capsule and computing $\sum p_{i}r_{i}$ , where *p_i_* is the pixel intensity and *r_i_* is the distance to the main cell axis.

The analyses were performed using R and the raw data along with the script used to analyze it and generate the figures are provided as Supplementary Dataset.

For the data presented in Fig. 3C, the pom1-as-tdTomato and corresponding pom1-GFP or pom1^KD^-GFP were extracted using Cellophane and smoothed using a Gaussian filter. The profile mid-point was the computed as the point on the profile at equal distance from the two poles. A measure of localization was defined as the log of the ratio between the sums of the pom1-as signal at the two poles and at the two mid-points. The GFP signal at mid-point was found to be the variable mostly correlated with that log-ratio. It was thus used as a covariate in a linear regression analysis including drug treatment as a binary variable and the log-ratio as explained variable. Four cells with Pom1^KD^-GFP signal at mid-point exceeding the (visually determined) saturation point were removed from the analysis.

In vitro kinase assay

GST-Pom1 fusion proteins were expressed in BL21 cells and purified with glutathione sepharose 4B (GE Healthcare) columns according to the manufacturer’s protocols. Kinase assays were performed in 30mM Tris, 100mM NaCl, 10mM MgCl2, 1mM EGTA, 10% glycerol, 20 mM ATP and 2 microCi [ϒ -32P]ATP (PerkinElmer #BLU502A250UC) in 15µl reaction mix as described (Hachet et al, 2011) with various concentrations of recombinant GST-Pom1, GST-Pom1^KD^ or GST-Pom1^(305-end) KD^, as indicated. The GST-Pom1^(305-end) KD^ allele was described in (Bhatia et al, 2013). After 30 min incubation at 30°C, the reaction was stopped by adding loading buffer and boiling 5 min at 95°C. Samples were diluted to appropriate concentration such that 40ng GST-Pom1 was loaded on a 6% SDS-PAGE gel, which was used to detect both the phosphorylation as well as loaded amounts, except for Fig. S7C, where duplicate gels were run. ^32^P-incorporation was detected with a phosphorimager and silver staining was performed using Pierce® Silver Stain Kit (Themo scientific).

**Legends of Supplementary Figures**

**Fig. S1: Total amount of Pom1 is not conserved across cells.**

A: The total (cytoplasmic and cortical) amount of Pom1 in each cell varies over a two-fold range. **B** Within a given cell, significant differences of Pom1 gradient amplitudes between the two poles can be observed. In about a third of the cells, this difference is more than two-fold. **C**. The amount of Pom1 in the cortex is correlated with the concentration of Pom1 in the cytoplasm. **D.** However, the amplitude of Pom1 at the pole does not show a correlation with the cytoplasmic Pom1 concentration. **E**. The ratio between cytoplasmic and cortical Pom1 is estimated to be between 0.6 and 1 for about two thirds of the cells and sometimes even greater than 1. **F.** The linear regression of the log-log plot of the total amount of cortical Pom1 vs the sum of the gradient amplitudes at the two poles results in a slope of 0.46 (black line, shaded area represents the 2SE confidence interval). This is consistent with the 0.5 power law predicted by the model between the total area under the gradient and the gradient amplitude (red line). Logarithms are in base 10.

All Pom1 quantification units are arbitrary (but consistent).

**Figure S2: Pom1 coefficient of variation decreases away from the cell tip**

The coefficient of variation of the 20 average profiles shown in Fig. 1C. It decreases along the cortex despite the expected decrease in signal to measurement noise ratio. This is indicative of a strong variation buffering mechanism between the pole and the center of the cell.

**Figure S3: Negative correlation between Pom1 gradient amplitude and decay length is predicted by either Pom1 clustering or Pom1 *trans*-phosphorylation**

The simple cluster-based model and the *trans*-phosphorylation model both predict a negative correlation between Pom1 gradient amplitude and decay length, and thus generate gradients that buffer variations in the gradient amplitude. The simulation data is the same as in Fig. 2C.

**Figure S4: A two-component cluster model predicts positive relationship between Pom1/Tea4 and Tea4**

The detailed cluster formation model of Saunders *et al.* (Saunders et al, 2012) predicts positive relationship between Pom1/Tea4 and Tea4, similar to our simple model of cluster formation. This model thus does not lead to buffering of Tea4 concentration at the pole. The vertical dashed line indicates the point corresponding to the parameters specified in Saunders *et al.*

**Figure S5: The negative relationship between Pom1/Tea4 and Tea4 is predicted by the *trans*-phosphorylation model, but not by the simple cluster-based model**

**A-B.** The same data as in Fig. 2B-C represented on a linear scale.

**Figure S6: Pom1 profiles can be fit by both exponential and power-law functions**

Each of the Pom1 profiles depicted in Fig. 1C was fitted to both an exponential function (obtained through linear regression in the log-lin space) and a power-law profile (obtained by linear regression in the log-log space). The fraction of explained variance (r^2^) of the linear regression was used to measure the quality of fit. In the graph, each dot represents a profile and is characterized by two r^2^ values corresponding to an exponential (x-axis) or a power-law (y-axis) fit. Dots above (respectively below) the red diagonal are fitted better (respectively worse) with a power law than an exponential. Both exponential and power law functions provide equally good fits, indicating that individual Pom1 profiles are of little help to distinguish between them.

**Figure S7. Pom1 intermolecular auto-phosphorylation in vitro.**

**A-B.** Two additional experiments, as in Figure 3A. Pom1 in vitro kinase assays with [ϒ-^32^P] ATP at five distinct Pom1 concentrations, ranging from 2.6 to 32ng/µl (1x to 12x). Loading of equivalent Pom1 amounts (40ng; see silver staining control) reveals higher incorporation of ^32^P upon higher Pom1 concentration, indicating an intermolecular reaction. Quantification is shown on the graphs below.

**C.** Pom1 in vitro kinase assays with [ϒ-^32^P] ATP at two distinct Pom1 concentrations (1x and 3x). Loading of equivalent Pom1 amounts (1X and 1/3X of reaction volume, respectively; see silver staining control) reveals higher incorporation of ^32^P upon higher Pom1 concentration, indicating an intermolecular reaction. Quantification is shown on the graph. The left part of the gel also shows lack of ^32^P incorporation upon use of inactive GST-Pom1^KD^ as control.

**D.** In vitro phosphorylation assay with [ϒ-^32^P] ATP of wt full-length Pom1 (Pom1^FL^) and inactive N-terminally truncated Pom1 (Pom1^(305-end) KD^). The inactive truncated Pom1 can serve as substrate for wt full-length Pom1.

**Supplementary Table S1: Strain list**

| **Strain number** | **Genotype** | **Source** |
| --- | --- | --- |
|  | **Figure 1** |  |
| YSM1276 | h+ pom1-tdTomato-natMX tea4-GFP-kanMX ade6-M216 leu1-32 ura4-D18 | ([Hachet et al, 2011](#_ENREF_13)) |
|  | **Figure 3** |  |
| YSM2418 | h+ pom1^as1^-dTtomato-natMX ade6-M216 leu1-32 ura4-D18 [pREP41 pom1-eGFP] | This study |
| YSM2419 | h+ pom1^as1^-dTtomato-natMX ade6-M216 leu1-32 ura4-D18 [pREP41-pom1^KD^-eGFP] | This study |

**References**

Bähler J, Wu JQ, Longtine MS, Shah NG, McKenzie A 3rd, Steever AB, Wach A, Philippsen P, Pringle JR (1998) Heterologous modules for efficient and versatile PCR-based gene targeting in Schizosaccharomyces pombe. *Yeast* 14: 943 – 951

Bendezu FO, Vincenzetti V, Martin SG (2012) Fission yeast Sec3 and Exo70 are transported on actin cables and localize the exocyst complex to cell poles*. PLoS ONE* 7: e40248

Moreno S, Klar A, Nurse P (1991) Molecular genetic analysis of fission yeast4Schizosaccharomyces pombe. *Methods Enzymol* 194: 795 – 823

Pan KZ, Saunders TE, Flor-Parra I, Howard M, Chang F (2014) Cortical regulation of cell size by a sizer cdr2p*. eLife* 3: e02040
