# Supplementary material for: Pom1 gradient buffering through intermolecular auto-phosphorylation
Source: Mol Syst Biol. 2015 Jul 6;11(7):818. doi: 10.15252/msb.20145996 (PMC4547846; doi:10.15252/msb.20145996)
Supplement: Supplementary file 10 [file msb0011-0818-sd10.pdf]

Supplementary Text S1
A model of Pom1 gradient formation through intermolecular auto-phophorylation

Micha Hersch, Sascha Dalessi, Olivier Hachet, Pranav Ullal, Payal Bhatia, Sophie G. Martin and Sven Bergmann*

1 The model

Pom1 is a kinase that can auto-phosphorylate on multiple sites and forms a diffusive gradient along the cortex of *S. pombe*. The gradient is initiated by de-phosphorylation of Pom1 at the cell poles, which allows Pom1 to bind the cortex. This de-phosphorylation is achieved by the Tea4-Dis2 phosphatase complex, which is actively brought to the cell poles by microtubules. At the cortex, Pom1 can diffuse laterally and re-phosphorylate. Upon each phosphorylation event, Pom1 decreases its affinity to the membrane, eventually detaching into the cytosol. Rapid diffusion in the cytosol allows its encounter and binding to the Tea4-Dis2 at the cell pole for the next round of dephosphorylation. In this section, we present a model of Pom1 gradient formation based on intermolecular phosphorylation of the Pom1 kinase. We start from a detailed model including the different phosphorylation states of Pom1 and show analytically that this model can be well approximated by a simple model involving super-linear decay.

Our model of Pom1 gradient formation assumes intermolecular phosphorylation of Pom1. We also assume a large number n of phosphorylation sites (at least 6 have been documented to be relevant for gradient formation [1]). We simplify the geometrical aspect of the problem by assuming a rotational symmetry of the cell. The cell shape is well described by a capsule, i.e., a cylinder with half spheres at the poles. However, we start by approximating the cell as a cylinder, allowing us to assume that the gradient takes place on a straight line. The impact of this assumption on the profile is negligible, as documented in Section 7.

Let $P(x,t)$ be the total amount of Pom1 at position x and time t, and $P_i(x,t)$ the amount of Pom1 phosphorylated i times. We thus have $\sum_0^n P_i = P$. We further assume that all P_i have the same diffusion and phosphorylation constants (respectively D and β), but different detachment rates κ_i that increase with the phosphorylation state i, with $\kappa_0 = 0$ [1]. We also assume, consistent with the data presented in [1], that the amount of phosphorylated Pom1 that reattaches

*The author order is not the same as in the main text to highlight the contributions specific to the theoretical analyses

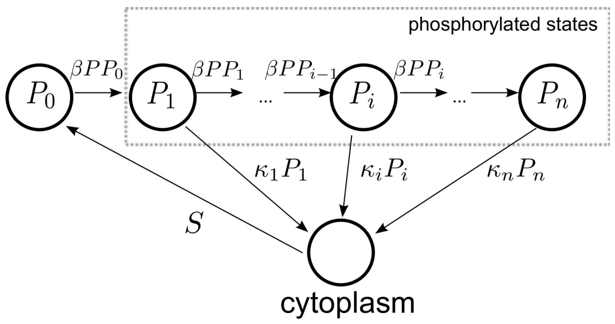

Figure 1: State representation of the Pom1 concentration. Arrow labels indicate the amount of Pom1 that undergo the corresponding state transition in a small time interval.

to the membrane is negligible. We therefore assume that Pom1 molecules are brought in non-phosphorylated state to the cortex at a rate $S(x,t)$ (which may depend on other factors such as the Pom1 concentration in the cytosol or the availabily of the Tea4-Dis2 complex). Pom1 dynamics can then be described as follows

$$\begin{aligned}
\frac{\partial P_0}{\partial t} &= D\frac{\partial^2 P_0}{\partial x^2} - \beta P P_0 + S \\
\frac{\partial P_1}{\partial t} &= D\frac{\partial^2 P_1}{\partial x^2} - \kappa_1 P_1 - \beta P P_1 + \beta P P_0 \\
&\vdots \\
\frac{\partial P_i}{\partial t} &= D\frac{\partial^2 P_i}{\partial x^2} - \kappa_i P_i - \beta P P_i + \beta P P_{i-1} \\
&\vdots \\
\frac{\partial P_n}{\partial t} &= D\frac{\partial^2 P_n}{\partial x^2} - \kappa_n P_n + \beta P P_{n-1}\,.
\end{aligned}$$

Summing those equations yields

$$\frac{\partial P}{\partial t} = D\frac{\partial^2 P}{\partial x^2} - \sum_{i=1}^{n} \kappa_i P_i + S. \tag{1}$$

In order to estimate the P_i, we assume for a moment that diffusion is small compared to detachment and phosphorylation rates, such that we can make the following adiabatic approximation: the various phosphorylation states of Pom1 at a given position are always in quasi steady-state neglecting the effect of diffusion. We can thus represent the phosphorylation and detachment dynamics with the state transitions depicted in Fig. 1. At steady state, the flows of molecules in and out of each state are equal such that we have:

$$\beta P P_{i-1} = \beta P P_i + \kappa_i P_i \quad \Rightarrow \quad P_i = \frac{\beta P}{\beta P + \kappa_i} P_{i-1} \quad \forall i \quad 0 < i < n\,. \tag{2}$$

It follows that

$$P_i = P_0 \prod_{j=1}^{i} \frac{\beta P}{\beta P + \kappa_j} = P_0 \prod_{j=1}^{i} \frac{1}{1 + \frac{\kappa_j}{\beta P}} \tag{3}$$

and we assume that the amount of Pom1 in the last state P_n is negligible as the κ_i increase with i. The overall detachment rate given by the sum term in (1) is then given by

$$\sum_{i=1}^{n} \kappa_i P_i = P_0 \sum_{i=1}^{n} \kappa_i \prod_{j=1}^{i} \frac{1}{1 + \frac{\kappa_j}{\beta P}} = \beta P_0 P \tag{4}$$

The last equation can be deduced by considering that at steady state, the rate of molecules entering and exiting the meta-state defined by the union of all phosphorylated states (see rectangle in Fig. 1) are equal.[1]
Now, using (3), we have

$$P = \sum_{i=0}^{n} P_i = P_0 \sum_{i=0}^{n} \prod_{j=1}^{i} \frac{1}{1 + \frac{\kappa_j}{\beta P}} \Rightarrow P_0 = \frac{P}{\sum_{i=0}^{n} \prod_{j=1}^{i} \frac{1}{1+\frac{\kappa_j}{\beta P}}} \, . \tag{5}$$

Given that κ_i increases with i and assuming $\kappa_0 = 0$, we can model the following relationship between κ_i and i

$$\kappa_i = \kappa i^c \, , \tag{6}$$

where c is a parameter quantifying how fast κ_i increases with i. It can be shown (see Appendix A), that if $\frac{\beta P}{\kappa}$ is large enough, we have

$$\sum_{i=0}^{n} \prod_{j=1}^{i} \frac{1}{1 + \frac{\kappa j^c}{\beta P}} \approx f(c) \big(\frac{\beta P}{\kappa}\big)^{\frac{1}{c+1}} \quad \text{with} \quad 1 \le f(c) = \frac{\Gamma(\frac{1}{c+1})}{(c+1)^{\frac{c}{c+1}}} < 1.5 \, , \tag{7}$$

where Γ is the standard Gamma function. Inserting this into (5) leads to

$$P_0 \approx \frac{1}{f(c)} \big(\frac{\kappa}{\beta}\big)^{\frac{1}{c+1}} P^{\frac{c}{c+1}} \, , \tag{8}$$

indicating that $P_0 \propto P^\eta$, with $\eta \le 1$.

Due the mixing caused by the actual diffusion, the relative amounts between P_0 and P tend to homogenize across the profile. A perfect mixing would indeed imply that $P_0 \propto P$, that is $\eta = 1$. Hence, $c/(c+1)$ must be considered a lower bound on the effective value of η.
We can thus rewrite (1) as

$$\frac{\partial P}{\partial t} = D \frac{\partial^2 P}{\partial x^2} - \alpha P^\gamma + S, \tag{9}$$

where $1 < \gamma = 1 + \eta \le 2$ and $\alpha = \beta P_0 / P^\eta$ can be seen as the effective detachment rate.

[1]It can also be shown more formally by the following formula $\sum_{i=0}^{n} a_i \prod_{j=1}^{i} \frac{1}{1+a_j} = 1 - \prod_{i=1}^{n} \frac{1}{1+a_i}$, which can be easily proven by induction on n and considering the product on the RHS being negligible for large enough n. We thank anonymous contributors to the math.stackexchange forum for pointing this out.

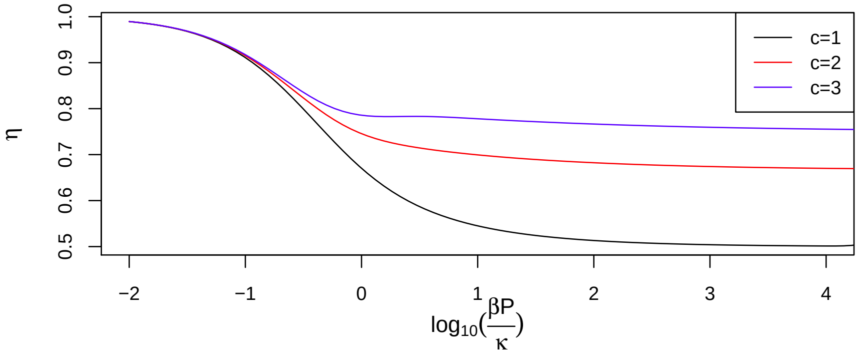

Figure 2: Convergence of P_0 to a power of P in (8) as the ratio $\beta P/\kappa$ increases for various levels of cooperativity indicated by c in (6). If βP is at least somewhat bigger than κ the approximation $P_0 \approx P^{\frac{c}{c+1}}$ is accurate.

A simple model where Pom1 detachment is caused by the repulsive Colomb forces between phosphate groups and the cell membrane (both negatively charged), would lead to $c \geq 1$ or $\eta \geq 1/2$ as the force increase linearly with the number of phosphorylated sites. Any kind of cooperativity between the phosphorylated sites for the membrane detachment would increase c and bring η closer to 1.

2 Gradient shape

In this section we describe an analytical solution to the simplified model given by (9), assuming that the source is localized at the cell pole. Discarding the domain of Pom1 attachment around the cell pole, the gradient is given by the solution of the following equation at steady-state

$$\frac{\partial P}{\partial t} = D\frac{\partial^2 P}{\partial x^2} - \alpha P^\gamma = 0\,. \tag{10}$$

Making the Ansatz that $P(x) = (ax+b)^c$, defining A as the amplitude of the gradient at the pole $A = P(0)$ and imposing $\lim_{x\to\infty} P(x) = 0$ because the cell length is large compared to the decay length, we get to the solution (see also [2])

$$P(x) = A\left(\sqrt{\frac{\alpha A}{2D(\gamma+1)}}(\gamma-1)x+1\right)^{-\frac{2}{\gamma-1}} = A\Big(\frac{x_0(\gamma)}{x+x_0(\gamma)}\Big)^{\frac{2}{\gamma-1}}\,, \tag{11}$$

with

$$x_0(\gamma) = \sqrt{\frac{2(\gamma+1)D}{(\gamma-1)^2\alpha A}}\,. \tag{12}$$

If we assume $\gamma = 2$, this becomes

$$P(x) = A\left(\sqrt{\frac{\alpha A}{6D}}x + 1\right)^{-2} = A\frac{x_0^2}{(x+x_0)^2} \quad \text{with} \quad x_0 = x_0(2) = \sqrt{\frac{6D}{\alpha A}}, \tag{13}$$

where x_0 can be thought of the natural length scale of the gradient (1.97 microns on average in our data).

3 Source quantification

We now turn to the source term S that was left unspecified in the above sections. It describes the attachment of non-phosphorylated Pom1 to the membrane at the cell poles. It is believed that the Tea4-Dis2 phosphatase complex is actively brought to the cell poles by the microtubules. It binds and de-phosphorylates cytoplasmic Pom1, which then binds to the membrane at the cell pole. As a first approximation, S can thus be assumed to be proportional to both the Tea4 concentration at the pole and the cytoplasmic Pom1 concentration.

Since the cell is a closed system and we assume a steady state gradient, the amount of Pom1 that attaches to the membrane from the cytoplasm is equal to the overall amount of Pom1 that detaches from the membrane into the cytoplasm. In other words, the rates of total attachment and detachment over the whole profile are equal. The total attachment S is thus given by

$$S = \int_0^\infty \alpha P(x)^\gamma \, \mathrm{d}x \tag{14}$$

$$= \int_0^\infty \alpha A^\gamma \left(\sqrt{\frac{\alpha A}{2D(\gamma+1)}}(\gamma-1)x + 1\right)^{-\frac{2\gamma}{\gamma-1}} \mathrm{d}x \tag{15}$$

$$= \frac{-\alpha A^\gamma(\gamma-1)}{\sqrt{\frac{\alpha A}{2D(\gamma+1)}}(\gamma-1)(\gamma+1)} \left[\left(\sqrt{\frac{\alpha A}{2D(\gamma+1)}}(\gamma-1)x + 1\right)^{-\frac{\gamma+1}{\gamma-1}}\right]_0^\infty \tag{16}$$

$$= \sqrt{\frac{2\alpha D}{\gamma+1}} A^{\gamma-\frac{1}{2}} \, . \tag{17}$$

If the number of Pom1 molecules attaching to the membrane is proportional to the Tea4 concentration, this relationship predicts the following power law between Tea4 and Pom1 at the pole:

$$A = (\sqrt{\frac{\gamma+1}{2\alpha D}}S)^{\frac{2}{2\gamma-1}} \overset{\text{if } \gamma=2}{=} \sqrt[3]{\frac{3}{2\alpha D}} S^{\frac{2}{3}} \, . \tag{18}$$

For $\gamma = 2$, this implies a 2/3 power law. Precisely this has been observed experimentally (see Figure 1 of the main text), confirming that γ is equal or close to two.

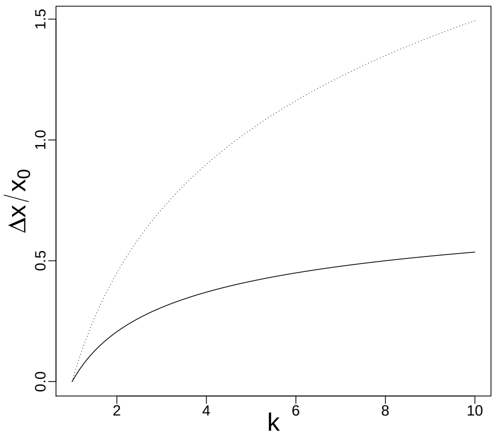

Figure 3: The expected shift in positional information as a function of the fold increase k in Pom1 attachment rate (solid line) compared to the corresponding shift in a non-buffered exponential gradient (dotted lines).

4 Buffering

Knowing the shape of the gradient profile, we can now quantify the buffering such a mechanism provides. Defining the decay length λ as the position at which the profile divides its amplitude by $e = 2.7\ldots$, we have:

$$P(\lambda) = \frac{A}{e} = A\Big(\sqrt{\frac{\alpha A}{2D(\gamma+1)}}(\gamma-1)\lambda + 1\Big)^{-\frac{2}{\gamma-1}}\,, \tag{19}$$

leading to

$$e = \Big(\sqrt{\frac{\alpha A}{2D(\gamma+1)}}(\gamma-1)\lambda + 1\Big)^{\frac{2}{\gamma-1}} \tag{20}$$

$$\Rightarrow \quad \lambda = \frac{\exp\left(\frac{\gamma-1}{2}\right) - 1}{\gamma - 1}\sqrt{\frac{2D(\gamma+1)}{\alpha A}} \overset{\text{if } \gamma=2}{=} (\sqrt{e} - 1)x_0\,. \tag{21}$$

The model thus predicts that the decay length decreases with the inverse square root of the amplitude at the pole. This has also been verified experimentally (see Figure 1 of the main text).

Following [3], we can compute the effect dx of a small variation dS in the source term S on the positional information

$$dx = \left|\left(\frac{\partial P}{\partial x}\right)^{-1}\frac{\partial P}{\partial S}\right| dS\,. \tag{22}$$

Inserting (18) into (9) and defining $a = \frac{1}{x_0 \sqrt[3]{S}} = \sqrt{\frac{\alpha}{6D}\sqrt{\frac{3}{2\alpha D}}} = \sqrt[4]{\frac{\alpha}{24D^3}}$, we have

$$\begin{aligned} P(x) &= \sqrt[3]{\frac{3}{2\alpha D}} S^{2/3} \big(a S^{1/3} x + 1 \big)^{-2} \\ \frac{\partial P}{\partial x} &= -2 \sqrt[3]{\frac{3}{2\alpha D}} a S (a S^{1/3} x + 1)^{-3} \\ \frac{\partial P}{\partial S} &= \sqrt[3]{\frac{3}{2\alpha D}} \big(-\frac{2}{3} a x (a S^{1/3} x + 1)^{-3} + \frac{2}{3} S^{-1/3} (a S^{1/3} x + 1)^{-2} \big) \\ \left(\frac{\partial P}{\partial x}\right)^{-1} \frac{\partial P}{\partial S} &= \frac{x}{3S} - \frac{\big(a S^{1/3} x + 1\big)^{-2}}{3 a S^{4/3}} = \frac{-1}{3 a S^{4/3}} \,. \end{aligned} \tag{23}$$

Local sensitivity to S is thus given by

$$dx = \sqrt[4]{\frac{24D^3}{\alpha}} \frac{dS}{3S^{\frac{4}{3}}} = \frac{x_0}{3} \frac{dS}{S} \,. \tag{24}$$

This means that the absolute imprecision on the position x is independent of x (as can be expected from a diffusive gradient) and increases slower than the relative imprecision on the Pom1 attachment rate $\frac{\Delta S}{S}$. The global sensitivity can then be computed by considering the difference in positional information Δx induced by multiplying the source term S_0 by a factor $k > 1$.

$$\Delta x = \int_{S_0}^{kS_0} \frac{S^{-\frac{4}{3}}}{3a} dS = -\frac{1}{a} \big[S^{-\frac{1}{3}} \big]_{S_0}^{kS_0} = -\big(\frac{1}{\sqrt[3]{k}} - 1\big) \frac{1}{a \sqrt[3]{S_0}} = \big(1 - \frac{1}{\sqrt[3]{k}}\big) x_0 \,. \tag{25}$$

This shows that x_0 constitutes an upper bound on Δx. Moreover, it implies that an overall higher attachment rate will translate into a more precise positional information, as x_0 can be reduced by bringing more Pom1 to the pole. As expected and illustrated in Fig. 3, Δx is smaller than for a non-buffered exponential profile, given by $(1 - \sqrt{e}) \log k$ [4]. A realistic upper value of $k = 8$ corresponds to a positional shift of about half of the length scale, which fits well with the observed variability of the Cdr2 domain boundary which has a standard deviation of about 30% of the largest length scale [5].

We can also consider two profiles of different amplitudes at the pole A_1 and A_2. Assuming $\gamma = 2$, at a distance x_L away from the pole the ratio between the concentrations of the two profiles P_1 and P_2 is given by

$$\frac{P_1(x_L)}{P_2(x_L)} = \frac{A_1}{A_2} \Big(\frac{\sqrt{\frac{\alpha A_2}{6D}} x_L + 1}{\sqrt{\frac{\alpha A_1}{6D}} x_L + 1} \Big)^2 \quad \to 1 \quad \text{if} \quad \sqrt{A_i} x_L >> \sqrt{\frac{6D}{\alpha}} \tag{26}$$

This indicates that high amplitudes gradients are buffered at a shorter range than low amplitude gradients and that reducing the diffusion constant (for example with a cluster formation mechanism) increases the phosphorylation-driven buffering.

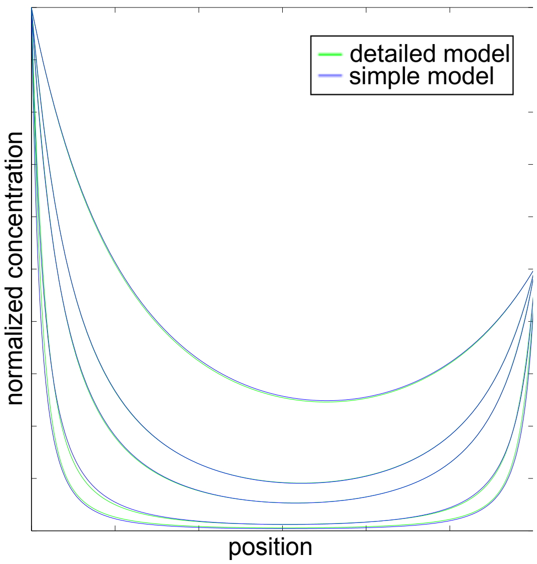

Figure 4: Numerical comparison between a detailed model and the simple model with squared detachment. The detachment rates have been set to $\kappa_i = i\kappa$ to represent the Coulomb forces presumably reducing the affinity of phosphorylated Pom1 to the membrane. The various curves correspond to various values of $\frac{\beta}{\kappa} = \{0.01, 0.05, 0.1, 0.5, 1\}$ from top to bottom.

5 Total Pom1 in gradient

The total amount of Pom1 in a gradient can be computed as the area under the gradient curve. This is given by

$$P_{tot} = \int_0^\infty P(x)\,\mathrm{d}x = \int_0^\infty A\left(\sqrt{\frac{\alpha A}{2D(\gamma+1)}}(\gamma-1)x+1\right)^{-\frac{2}{\gamma-1}} \mathrm{d}x \tag{27}$$

$$= \frac{A(\gamma-1)}{\sqrt{\frac{\alpha A}{2D(\gamma+1)}}(\gamma-1)(\gamma-3)}\left[\left(\sqrt{\frac{\alpha A}{2D(\gamma+1)}}(\gamma-1)x+1\right)^{\frac{\gamma-3}{\gamma-1}}\right]_0^\infty \tag{28}$$

$$= \sqrt{\frac{2(\gamma+1)D}{(\gamma-3)^2\alpha}}A^{\frac{1}{2}} = \frac{\gamma-1}{3-\gamma}x_0 A \stackrel{\text{if } \gamma=2}{=} x_0 A \tag{29}$$

$$\Rightarrow A = \frac{(3-\gamma)^2\alpha}{2(\gamma+1)D}P_{tot}^2 \tag{30}$$

If the gradient extends to a large enough range such that it can be approximated by infinity, a square power law between the total amount of Pom1 and the amplitude at the pole is expected and consistent with our experimental data (see Supplemental Fig. S1F). Simulations indicate that such an approximation can be made only for ranges bigger than λ (or x_0) by at least an order of magnitude. For smaller ranges the power law is between 1 and 2.

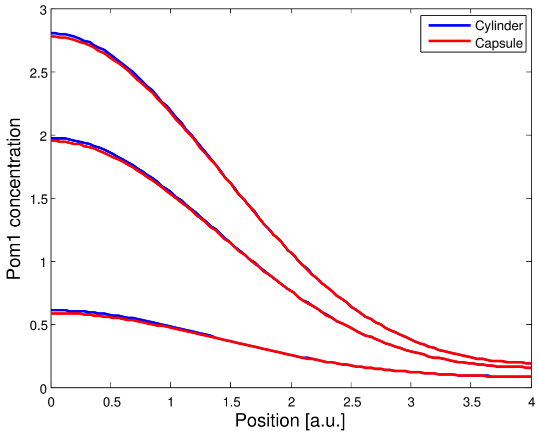

Figure 5: The profiles obtained when approximating the cell with a cylinder or a capsule are very similar. The three curves correspond to different values of S.

6 Numerical validation

In addition to experimental evidence, numerical simulations were performed to verify that violations of the assumptions described above do not hamper the validity of the model. Using the standard Matlab numerical solver and assuming six phosphorylation sites, the gradients were generated using (1), for various values of α/κ. The diffusion constant D is not a free parameter of the model, and was set to one. Using a Gaussian Adaptation optimizer [6], the α parameter was then optimized to match the simple trans-phosphorylation model, i.e., (9) with $\gamma = 2$. The results, shown in Fig 4 indicate that the simple model provides a good approximation of the detailed model, for the tested gradient shapes.

7 Cell geometry

In the above model, the cell was assumed to be a cylinder to allow an analytical solutions to the model. We show here that this assumption has a minimal impact on the results. In (9), the only term that is affected by the cell geometry is the diffusion term $D\frac{\partial^2 P}{\partial x^2}$. We thus consider diffusion at the cell tip as diffusion on a sphere. The diffusion term is given by the divergence of the flux $\mathbf{J}$ of P. Considering the standard spherical coordinates defined by (r, θ, ϕ), we have

$$\operatorname{div}\mathbf{J} = \frac{1}{r^2}\frac{\partial}{\partial r}(r^2\mathbf{J}_r) + \frac{1}{r\sin\theta}\frac{\partial}{\partial\theta}(\sin\theta\mathbf{J}_\theta) + \frac{1}{r\sin\theta}\frac{\partial\mathbf{J}_\phi}{\partial\phi}\,, \tag{31}$$

where $\mathbf{J}_\theta = \frac{D\partial P}{r\partial\theta}$, and $\mathbf{J}_r = \mathbf{J}_\phi = 0$ because the diffusion is limited to the cortex and because of rotational symmetry. Hence

$$\operatorname{div}\mathbf{J} = \frac{1}{r\sin\theta}\frac{\partial}{\partial\theta}(\sin\theta\mathbf{J}_\theta) = \frac{D}{r^2}(\frac{1}{\tan\theta}\frac{\partial P}{\partial\theta} + \frac{\partial^2 P}{\partial\theta^2}) = D(\frac{1}{r\tan\frac{x}{r}}\frac{\partial P}{\partial x} + \frac{\partial^2 P}{\partial x^2})\,, \tag{32}$$

	Source of variations across cells	Constant across cells	Predicted slope between log A and log λ
1	Diffusion D	$P_{tot} = \frac{k(P_c)T}{\alpha}$	-1
2	Detachment rate α	$\frac{k(P_c)T}{\alpha}$	-1
3	D and α	$\frac{k(P_c)TD^{1/2}}{\alpha^{3/2}}$	-1/2
4	Pom1 attachment rate $k(P_c)T$	α, D	-1/2

Table 1: Various hypotheses for a negative correlation between Pom1 gradient amplitude A and decay length λ. The top three rows correspond to cases of linear detachment and the last row corresponds to our supra-linear detachment hypothesis. Unlike the total cortical Pom1, which varies across cells, the diffusivity and affinity of a Pom1 molecule to the membraine are very likely to be constant across cells. The top three hypotheses can thus be safely rejected. Furthermore, as shown in line 3, the coordination between the varying parameters required to account for a -1/2 power law seems not realistic.

because $x = r\theta$. As can shown in Fig. 5, using this last expression for the diffusion in the spherical part of the cortex produces very similar profiles than the ones obtained with the cylindrical approximation. In those simulations and consistently with the experimental observations, a Gaussian source was considered with a standard deviation set equal to the radius of the sphere. This confirms that our cylindrical approximation, which is necessary for the derivation of the analytical solutions, is justified. As for the point source approximation, it has been previously shown to only impact the solution close to the source [7].

8 Aternative hypothesis

It could be argued that the decreasing relationship between the Pom1 gradient amplitude and its decay length is not due to a buffering mechanism but rather to a very tight control of cortical Pom1 that ensures it remains constant across cells.

Assuming a linear decay, we have:

$$\frac{\partial P(x,t)}{\partial t} = D\frac{\partial^2 P(x,t)}{\partial x^2} - \alpha P(x,t) + S(x,t) \qquad (33)$$

$$\frac{\partial P_c(t)}{\partial t} = \int_x \alpha P - S(x,t)dx\,, \qquad (34)$$

where S is likely to depend on the cytoplasmic Pom1 concentration P_c. This assumes that the production and degradation of Pom1 are negligible and Pom1 only cycles between the cortex and the cytoplasm. Assuming a point source $S(x,t) = S\delta(x)$ and solving (33) for steady state results in the classical exponential gradient

$$P(x) = Ae^{-\frac{x}{\lambda}} \quad \text{with} \quad \lambda = \sqrt{\frac{D}{\alpha}}\,. \qquad (35)$$

The relationship between A and S can be found either by considering (34) at steady state or equivalently by integrating (33) over x at steady state. The result for a point source is:

$$S = \int_x \alpha P dx = \alpha\lambda A = \alpha P_{tot} \Rightarrow A = \frac{S}{\alpha\lambda} = \frac{S\lambda}{D} = \frac{S}{\sqrt{D\alpha}} = \frac{P_{tot}}{\lambda}, \qquad (36)$$

where P_{tot} is the total cortical Pom1 over the profile. A reasonable hypothesis for S would assume that it is proportional to both the Tea4 concentration T at the pole and the cytoplasmic Pom1 $S = kP_cT$. However, our data (see Supplementary Figure S1) indicate that A is highly correlated with T but not with P_c, maybe because variations in T are much larger than variations in P_c or the reaction takes place at P_c saturation level. So we may remain general and write that $S = k(P_c)T$ and restrain from any assumption on the potential role of P_c in the Pom1 attachment rate.

Table 1 first lists three hypotheses explaining the observed buffering within the framework of a linear detachment and homogeneous diffusion. Those hypotheses are very unlikely as they assume that the variability in gradient shapes originate in the variability in Pom1 diffusion or detachment rate while maintaining a tight control of the total cortical Pom1 across cells. This is not consistent with our data, which display a large variability in total cortical Pom1 across cells. On the contrary our hypothesis assumes constant Pom1 properties across cells and sees the Pom1 attachment rate to the membrane as the main source of variability across cell (hypothesis 4 in Table 1). This is very likely to be the case given the known stochasticity of microtubule dynamics even within the same cell.

A Proof of equation (7)

Proposition[2]

$$\lim_{a\to\infty} a^{-\frac{1}{c+1}} \sum_{n=1}^{\infty} \prod_{k=1}^{n} \frac{a}{a+k^c} = \frac{1}{(c+1)^{\frac{c}{c+1}}} \Gamma(\frac{1}{c+1}) \quad \forall c > 0\,, \qquad (37)$$

where Γ is the standard Gamma function.

Proof Let us define $a = u^{c+1}$ and call the above sum s. Then we get

$$s = s(c,a) := a^{-1/(c+1)} \sum_{n=1}^{\infty} \prod_{k=1}^{n} \frac{a}{a+k^c} = \sum_{n=1}^{\infty} \prod_{k=1}^{n} \frac{1}{1+(k^c/u^{c+1})} \frac{1}{u}.$$

The main idea of the proof is the observation that the last infinite series above behaves like a Riemann sum as $u \to \infty$. To this end, we fix $M > 0$ and divide the sum into two parts s_1 and s_2 as defined below:

$$s = \sum_{n \le Mu} \prod_{k=1}^{n} \frac{1}{1+(k^c/u^{c+1})} \frac{1}{u} + \sum_{n > Mu} \prod_{k=1}^{n} \frac{1}{1+(k^c/u^{c+1})} \frac{1}{u} = s_1 + s_2.$$

[2]This proof was kindly provided by an anonymous contributor to the math.stackexchange forum.

First, let $N = \lfloor Mu \rfloor$. If $n > Mu$, then $n > N$ and

$$\prod_{k=1}^{n} \frac{1}{1+(k^c/u^{c+1})} \le \prod_{k=N+1}^{n} \frac{1}{1+(k^c/u^{c+1})}$$
$$\le \prod_{k=N+1}^{n} \frac{1}{1+M^c/u} = \left(\frac{1}{1+M^c/u}\right)^{n-N}.$$

This shows that

$$s_2 \le \frac{1}{u}\sum_{n=1}^{\infty}\left(\frac{1}{1+M^c/u}\right)^n = \frac{1}{M^c}.$$

Next, if $n \le N$ then

$$\begin{aligned}\log \prod_{k=1}^{n} \frac{1}{1+(k^c/u^{c+1})} &= -\sum_{k=1}^{n}\log\left(1+\frac{k^c}{u^{c+1}}\right)\\ &= -\int_0^n \log\left(1+\frac{t^c}{u^{c+1}}\right)\,dt + \mathcal{O}\left(\tfrac{M^c}{u}\right)\\ &= -\int_0^{n/u} u\log\left(1+\frac{t^c}{u}\right)\,dt + \mathcal{O}\left(\tfrac{M^c}{u}\right)\\ &= -\frac{(n/u)^{c+1}}{c+1} + \mathcal{O}\left(\tfrac{M^{2c+1}}{u}\right).\end{aligned}$$

Plugging this back,

$$s_1 = \sum_{n\le Mu}\left(1+\mathcal{O}\left(\tfrac{M^{2c+1}}{u}\right)\right)\exp\left(-\frac{(n/u)^{c+1}}{c+1}\right)\frac{1}{u}.$$

Taking $u \to \infty$, it follows that

$$\lim_{u\to\infty} s_1 = \int_0^M e^{-x^{c+1}/(c+1)}\,dx.$$

Combining two observations, we obtain

$$\int_0^M e^{-x^{c+1}/(c+1)}\,dx - \frac{1}{M^c} \le \liminf_{u\to\infty} s \le \limsup_{u\to\infty} s \le \int_0^M e^{-x^{c+1}/(c+1)}\,dx + \frac{1}{M^c}.$$

Taking $M \to \infty$ we finally obtain that $f(c) = \lim s$ exists and is equal to

$$f(c) = \int_0^\infty e^{-x^{c+1}/(c+1)}\,dx = \frac{1}{(c+1)^{\frac{c}{c+1}}}\Gamma(\frac{1}{c+1}).$$

References

[1] Olivier Hachet, Martine Berthelot-Grosjean, Kyriakos Kokkoris, Vincent Vincenzetti, Josselin Moosbrugger, and Sophie G. Martin. A phosphorylation cycle shapes gradients of the DYRK family kinase pom1 at the plasma membrane. *Cell*, 145(7):11161128, 2011.

[2] Avigdor Eldar, Dalia Rosin, Ben-Zion Shilo, and Naama Barkai. Self-enhanced ligand degradation underlies robustness of morphogen gradients. *Developmental cell*, 5(4):635–646, 2003.

[3] Aitana Morton de Lachapelle and Sven Bergmann. Precision and scaling in morphogen gradient read-out. *Molecular Systems Biology*, 6, March 2010.

[4] Sven Bergmann, Oded Sandler, Hila Sberro, Sara Shnider, Eyal Schejter, Ben-Zion Shilo, and Naama Barkai. Pre-steady-state decoding of the bicoid morphogen gradient. *PLoS biology*, 5(2):e46, 2007.

[5] Payal Bhatia, Olivier Hachet, Micha Hersch, Sergio A Rincon, Martine Berthelot-Grosjean, Sascha Dalessi, Laetitia Basterra, Sven Bergmann, Anne Paoletti, and Sophie G Martin. Distinct levels in pom1 gradients limit cdr2 activity and localization to time and position division. *Cell cycle*, 13(4):538–552, 2014. Re-analysis of published data.

[6] Christian L Müller and Ivo F Sbalzarini. Gaussian adaptation revisited–an entropic view on covariance matrix adaptation. In *Applications of Evolutionary Computation*, pages 432–441. Springer, 2010.

[7] S Dalessi, A Neves, and S Bergmann. Modeling morphogen gradient formation from arbitrary realistically shaped sources. *Journal of Theoretical Biology*, 294:130–138, 2012.
