# Supplementary material for: Pom1 gradient buffering through intermolecular auto-phosphorylation
Source: Mol Syst Biol. 2015 Jul 6;11(7):818. doi: 10.15252/msb.20145996 (PMC4547846; doi:10.15252/msb.20145996)
Supplement: Supplementary file 11 [file msb0011-0818-sd11.pdf]

Pom1 gradient buffering through intermolecular auto-phosphorylation

Micha Hersch, Olivier Hachet, Sascha Dalessi, Pranav Ullal, Payal Bhatia, Sven Bergmann and Sophie G. Martin

Corresponding author: Sophie G. Martin, University of Lausanne

Review timeline:

Submission date:	19 December 2014
Editorial Decision:	12 January 2015
Revision received:	20 February 2015
Editorial Decision:	18 May 2015
Revision received:	30 April 2015
Accepted:	04 June 2015

Editor: Thomas Lemberger

Transaction Report:

(Note: With the exception of the correction of typographical or spelling errors that could be a source of ambiguity, letters and reports are not edited. The original formatting of letters and referee reports may not be reflected in this compilation.)

1st Editorial Decision 12 January 2015

Thank you again for submitting your work to Molecular Systems Biology. We have now heard back from the three referees who agreed to evaluate your manuscript. As you will see from the reports below, the referees find the topic of your study of potential interest. They raise, however, several concerns which should be convincingly addressed in a revision of this work.

Without repeating all the points raised by the reviewers, the major issues refer to the following points:

- The observed relationship between gradient decay length and amplitude should be compared to the relationship that would be expected in absence of regulation. In other words, the observed slope of -1/2 should be compared to the slope -1, according to the reasoning provided by reviewer #1. This should be both evaluated statistically and discussed when interpreting the results. Crucially, the consequence of a slope -1/2 or -1 on the robustness in *positional information* to fluctuations in gradient amplitude should be explicitly described (ie what is the optimal relationship between A and lambda for minimizing fluctuation in positional information and how does it relate to the reported mechanism).

- measurement of the fraction of cortical vs cytoplasmic pom1 would be necessary and the conservation of the pom1p molecules should be taken into account in the model.

- reviewer #2 is asking for more direct evidence for the trans-phosphorylation mechanism and provides constructive suggestion in this regard.

© European Molecular Biology Organization

Reviewer #1:

The authors study the gradient of pom1p that forms on the inner plasma membrane of S.pombe cells. This is a good example of morphogen gradient happening in a amenable organism, and thus the study should be interesting to a wide audience. I found myself the problem very interesting, and I read the manuscript with care. I was really disappointed by the overall emphasis. A good quantitative match can be obtained, but I however quickly stumbled on a fundamental problem with the basic assumptions on which the story is built, as explained below. I believe it can be addressed without too much efforts and while this may lead to an important shift in the overall presentation of the material, but I expect that it will make the article much more pertinent. I hope that the authors will agree to reformulate their report, and my recommendation would be to accept the article for publication if these concerns are addressed.

The core of the research is the robustness of an intracellular gradient, and the particular point is to understand how the amplitude of this gradient is related to its length-scale. This gradient is described as an exponential A*exp(-lambda*x), and the authors look at the correlations between the two parameters A and lambda (eg. figure 1). It is implicitly assumed that A and lambda should be uncorrelated. This allow them to deduce, from the observed correlation, that some kind of 'buffering' exists in the system. They then present a plausible buffering mechanism, develop a mathematical model and present some experimental evidence to support it.

However, in my understanding, Tea1 and pom1p auto-phosphorylation affect the phosphorylation state of pom1p, but not the level of the molecule in the cell. This imply that the number of Pom1 molecules should remain constant in the system. For a simple 1D gradient, this means mathematically that the integral of A*exp(-lambda*x), between 0 and the cell length L must be equal to the total number of molecules P in the system, which is fixed. Assuming L is larger than 1/lambda, one can derive from this that A = lambda * P. Thus the amplitude A should be inversely proportional to the decay length (1/lambda).

In the context of the submitted work, this means that I would expect on Figure 1E a slope of -1, in the absence of any special molecular mechanism in the cell. Thus instead of comparing the observed slope of -1/2 to a slope of zero (the case with no correlation), one should compare with a slope of -1. The observed slope of -1/2 will need to be re-interpreted, in light of this. In any log-log plot (Figure 1E, F, 2B/C), I would strongly suggest using equal axis magnification in X and Y, such as to make the slopes -1, or -1/2 more readily visible.

I have above used the fact that the number of molecules in the gradient should be constant to derive the relation between A and lambda. The situation of pom1p in the S.pombe cell is not necessarily as simple, because there is a fraction of pom1p in the cytoplasm, and a faction on the plasma membrane. The cortical fraction is the one that forms a gradient. Hence, it will be essential for the revision to measure the relative amount of pom1p in the cytoplasm vs. on the cortex. If the cytoplasmic pool is small, then my criticism will hold fully. If the cytoplasmic pool would be much greater, the conservation in the number of molecules could possibly lead to a slope that is less negative than -1. From the images on Figure 3B, and without quantification, I am driven to conclude that the cytoplasmic pool is not large. This point needs to be answered by measurements.

The same concern should be addressed in the model. Specifically, the author should explicitly take into account the conservation of the pom1p molecules in the equations, by including a variable to represent the cytoplasmic fraction. The authors will then be able to contemplate the results shown in Figure 1F again, with a revised model that contains the basic amount of buffering provided by the conservation of Pom1p. I expect that, the article will need to be revised substantially. In any case, it will be important to justify the statements relating the "molecular buffering mechanism" by comparing two conditions in the model: in the presence and in the absence of the putative molecular activity. This should hopefully avoid the mistake of making statements based on incorrect/unjustified intuition. Considering what I said, I would not be surprised if instead of buffering the variations, there would be a mechanism to amplify them, and some of the interpretative statements may need to be inverted.

Another essential verification of the model is to compare the data presented on Figure 3A with the prediction of the detailed model of pom1p phosphorylation. It should be simple to verify if the model can reproduce the relative phosphorylation signal as a function of the concentration of pom1p. This is important to check some of the assumptions of the model, for example that phosphorylation rate beta is independent of the phosphorylation state. I would have myself assumed this phosphorylation rate to be proportional to the amount of remaining available sites on the proteins (n-i). A justification of this assumption is needed.

MODEL (Supplementary Text S1):

Figure 1 is missing the detachment of rate for P0, which goes back to the cytoplasm with k0*P0.

The overall scheme of chemical reactions is a-priori not thermodynamically consistent. If the release of Pom1 from the cortex occurs passively as described (i.e. from changes in electrostatic interactions), then one would also expects cytoplasmic unbound phosphorylated Pom1 to be able to rebind directly to the cortex. Thus for every species there should be a equilibrium, with two reverse reactions. It may be of course possible to deduce that the binding fluxes are small, of to argue in this direction in some way.

In equation (4) it seems that the sum for i should start at one and not zero, or S should appear in the equation. If was not clear how equation (4) is used in the rest of the analysis.

In "1. Gradient shape", applying boundary conditions is not valid here. Instead, one may use conservation of mass (the number of pom1p) to set the amplitude coefficients. The assumption of an infinite cell length should hold, but it can be justified more rigorously (L*lambda greater than 1).

In "2. Source quantification". The assumption that the number of Pom1 molecules attaching to the membrane is proportional to the Tea4 concentration violates basic chemical kinetic theory. This rate should also depend on the concentration of Pom1 in the cytoplasm. Conservation of Pom1 imply that the cytoplasmic concentration is inversely correlated with the total amount of Pom1 on the cortex. The cell is a closed system and this automatically provides a certain amount of 'buffering'. This important point concerns all the results of the paper.

Overall, the Supplementary Text S1 should be clarified by adding a justification for each part. In its present form, it looks like an exercise in algebra. One should state the assumptions, declare the result to be proven, before proceeding with the proof. Finally, it is important to first discuss a model based on the laws of chemistry, even if one can justify assumptions to depart from it.

Reviewer #2:

This manuscript examines how the protein kinase Pom1 forms a spatial gradient at the plasma membrane of fission yeast cells. These authors previously showed that Pom1 autophosphorylation is a key step in gradient formation because it controls Pom1-membrane interactions (Hachet et al., Cell, 2011). This mechanism was included in a subsequent mathematical model for gradient assembly published by others (Saunders et al., 2012). A key step in this previous model was formation of dynamic clusters, which were proposed to allow robust gradient assembly in the presence of Pom1 concentration fluctuations. In this current manuscript, the authors add one new conceptual component to a model for Pom1 gradient assembly. Specifically, autophosphorylation can occur through intramolecular/cis phosphorylation, or alternatively through intermolecular/trans phosphorylation. The distinction is important because intramolecular autophoshorylation should be concentration independent, while the rate/extent of intermolecular autophosphorylation will depend on protein concentration. This has major implications in a system like the Pom1 gradient, where the protein diffuses away from a point source as a dephosphorylated species. In my view, this is an extremely important concept that should be considered key to how this gradient forms, with relevance to how gradients form in other biological systems as well. The paper contains a combination of cell biology, biochemistry, and mathematical modeling to make this point. I have only minor comments that might be considered to strengthen the manuscript.

1. The in vitro case for trans-autophosphorylation (as opposed to cis) could be strengthened. The

concentration dependence shown in Figure 3A is consistent with trans phosphorylation, with additional contributions from cis as well. However, the authors could clearly show trans phosphorylation in vitro using purified Pom1 and GST-Pom1(kinase-dead). In the trans model, active wildtype protein will phosphorylate the purified kinase-dead protein. Adding something like a GST tag should move the kinase-dead protein to a region of the gel that separates it from the wildtype protein.

2. The data support a role for trans autophosphorylation but do not exclude a role for cis autophosphorylation. The authors should clearly state in the text that their model does not exclude the presence or potential role of cis-phosphorylation in the system. Since trans versus auto phosphorylation should compete for the same sites on Pom1, I also wonder if the authors can use their model to estimate the amount of trans versus cis in cells.

1st Revision - authors' response 20 February 2015

(see next page)

© European Molecular Biology Organization

Response to the reviewers' comments

Reviewer 1

The authors study the gradient of pom1p that forms on the inner plasma membrane of S.pombe cells. This is a good example of morphogen gradient happening in a amenable organism, and thus the study should be interesting to a wide audience. I found myself the problem very interesting, and I read the manuscript with care. I was really disappointed by the overall emphasis. A good quantitative match can be obtained, but I however quickly stumbled on a fundamental problem with the basic assumptions on which the story is built, as explained below. I believe it can be addressed without too much efforts and while this may lead to an important shift in the overall presentation of the material, but I expect that it will make the article much more pertinent. I hope that the authors will agree to reformulate their report, and my recommendation would be to accept the article for publication if these concerns are addressed.

*The core of the research is the robustness of an intracellular gradient, and the particular point is to understand how the amplitude of this gradient is related to its length-scale. This gradient is described as an exponential A*exp(-lambda*x), and the authors look at the correlations between the two parameters A and lambda (eg. figure 1). It is implicitly assumed that A and lambda should be uncorrelated. This allow them to deduce, from the observed correlation, that some kind of 'buffering' exists in the system. They then present a plausible buffering mechanism, develop a mathematical model and present some experimental evidence to support it.*

*However, in my understanding, Tea1 and pom1p auto-phosphorylation affect the phosphorylation state of pom1p, but not the level of the molecule in the cell. This imply that the number of Pom1 molecules should remain constant in the system. For a simple 1D gradient, this means mathematically that the integral of A*exp(-lambda*x), between 0 and the cell length L must be equal to the total number of molecules P in the system, which is fixed. Assuming L is larger than 1/lambda, one can derive from this that A = lambda * P. Thus the amplitude A should be inversely proportional to the decay length (1/lambda). In the context of the submitted work, this means that I would expect on Figure 1E a slope of -1, in the absence of any special molecular mechanism in the cell. Thus instead of comparing the observed slope of -1/2 to a slope of zero (the case with no correlation), one should compare with a slope of -1. The observed slope of -1/2 will need to be re-interpreted, in light of this.*

We thank the reviewer for attracting our attention to this aspect of the model, which we admittedly did not think about as we were considering the mechanism within the framework set by Saunders et al, 2012. Within a relatively short time interval within the cell cycle one may indeed assume that *P*, the total number of Pom1 molecules in a given cell does not change drastically (although one should not forget that across the entire cell cycle this number has to be doubled in order to achieve similar amounts of Pom1 in the daughter cells). Yet, importantly, our observation of a negative correlation between the amplitude *A* and the decay length *lambda* was made not within single cells but over a

population of cells. We apologize if this was not made clear enough in our text. In this context, an inversely proportional relationship between *A* and *lambda* on the basis of a constant *P* would have to assume that *P* does not change *across* cells. We examined whether this assumption is supported by our data and it appears not to be the case. On the contrary our data display a strong correlation between the area under the gradient and its amplitude and this correlation matches well a 3/2 power law predicted by our inter-molecular phosphorylation model. We added a supplementary Figure S1 and modified the main text to mention this hypothesis:

> "Variations in cortical amounts of Pom1 across cells indicate *that* this negative correlation cannot be explained by a mechanism that would keep the total amount of cortical Pom1 (and thus the area under the profile) constant across cells (Fig S1)."

We also added a section in the Supplemental Text investigating in more details how a negative power law between *A* and *lambda* could be obtained in the framework of homogeneous diffusion and linear detachment. We show that in principle a negative power of -1 could be achieved if the total Pom1 concentration was constant across cells, while the diffusion constant would vary between cells. To obtain a power of -1/2 would require a very tight coordination of the intra cellular variation in the diffusion constant and that in the detachment rate. Since these are a priori unrelated *molecular* properties of Pom1, we cannot think of a plausible explanation for such a dependency. In contrast, we find it reasonable to assume that the dominating source of variability is in the rate at which Pom1 is deposited at the cortex, since this is governed by the complex *cellular* process of microtubule-mediated transport, which is known to be of stochastic nature.

In any log-log plot (Figure 1E, F, 2B/C), I would strongly suggest using equal axis magnification in X and Y, such as to make the slopes -1, or -1/2 more readily visible.

We changed those plots accordingly.

I have above used the fact that the number of molecules in the gradient should be constant to derive the relation between A and lambda. The situation of pom1p in the S.pombe cell is not necessarily as simple, because there is a fraction of pom1p in the cytoplasm, and a faction on the plasma membrane. The cortical fraction is the one that forms a gradient. Hence, it will be essential for the revision to measure the relative amount of pom1p in the cytoplasm vs. on the cortex. If the cytoplasmic pool is small, then my criticism will hold fully. If the cytoplasmic pool would be much greater, the conservation in the number of molecules could possibly lead to a slope that is less negative than -1. From the images on Figure 3B, and without quantification, I am driven to conclude that the cytoplasmic pool is not large. This point needs to be answered by measurements.

We measured the relative amount of cortical vs cytoplasmic Pom1, which is now shown in Fig. S1. Our estimate indicates that cytoplasmic Pom1 corresponds to roughly half of cortical Pom1, so it is far from negligible. However, as mentioned above, in light of our observation that total Pom1 is not conserved across cells, this fact does not help explaining our observed dependence between *A* and *lambda* in the way suggested by the reviewer.

The same concern should be addressed in the model. Specifically, the author should explicitly take into account the conservation of the pom1p molecules in the equations, by including a variable to represent the cytoplasmic fraction. The authors will then be able to contemplate the results shown in Figure 1F again, with a revised model that contains the basic amount of buffering provided by the conservation of Pom1p. I expect that, the article will need to be revised substantially. In any case, it will be important to justify the statements relating the "molecular buffering mechanism" by comparing two conditions in the model: in the presence and in the absence of the putative molecular activity. This should hopefully avoid the mistake of making statements based on incorrect/unjustified intuition. Considering what I said, I would not be surprised if instead of buffering the variations, there would be a mechanism to amplify them, and some of the interpretative statements may need to be inverted.

The cytoplasmic Pom1 is now included in the model described in Section 7 of the supplemental text. In the original model, the conservation of Pom1 within one cell is implicitly included in the steady-state assumption. Indeed, the number of Pom1 molecules that reach the cortex from the cytoplasm equals the number of molecules that detach from the cortex to the cytoplasm, otherwise it is not at steady state (see equations (14) and (30)).

We strongly believe that our conclusions regarding buffering remain valid and the reduced variability of Pom1 vs Tea4 amplitude at the cell tip precludes the possibility that variations are amplified.

Another essential verification of the model is to compare the data presented on Figure 3A with the prediction of the detailed model of pom1p phosphorylation. It should be simple to verify if the model can reproduce the relative phosphorylation signal as a function of the concentration of pom1p. This is important to check some of the assumptions of the model, for example that phosphorylation rate beta is independent of the phosphorylation state. I would have myself assumed this phosphorylation rate to be proportional to the amount of remaining available sites on the proteins (n-i). A justification of this assumption is needed.

We thank the reviewer for this interesting suggestion. This *in vitro* experiment is somewhat different from what happens in vivo in the sense that *in vivo*, unlike *in vitro*, Pom1 is in a permanent cycle of phosphorylation-detachment-dephorylation. We thus adapted our model to account for this difference and fitted the data of Fig. 3A to the inter-molecular phosphorylation model. We found a good agreement, indicating that these data are at least compatible with it (see Fig A below). We further used this model to follow the suggestion of reviewer 2 and test the model for the importance of phosphorylation in *cis*. For the data presented in Fig. 3A, adding cis-phosphorylation did not notably improve the quality of the fit. However, this finding did not replicate for the fit of one of the replicate experiments shown in Fig S7 (that of S7B, shown in Fig A below), leaving us unable to draw a conclusion regarding the (possibly variable) importance of *cis*-phosphorylation. Because it is inconclusive regarding the contribution of cis-phosphorylation, we thus present this analysis here, but prefer not to include it in the manuscript where we simply comment that

"This, however, does not exclude that Pom1 also auto-phosphorylates intra-molecularly."

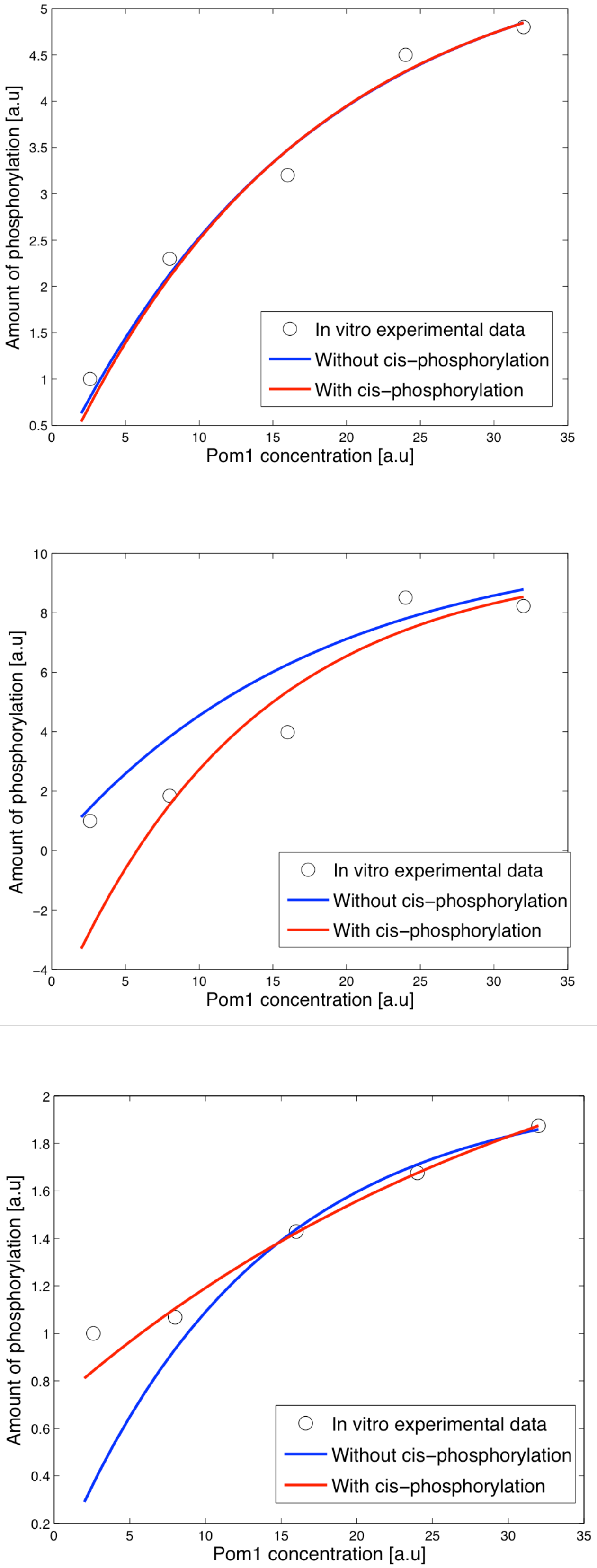

Fig A: In vitro experiments are quantitatively compatible with inter-molecular phosphorylation model

The data presented in Fig 3A (top), S7A (middle) and S7B (bottom) were fitted to the following model of intermolecular phosphorylation: $\acute{r} = \beta P(nP - r)$, where r is the observed phosphorylation signal, P the Pom1 concentration, n the number of phosphorylation sites per Pom1 molecule, and β is the inter-molecular phosphorylation rate. So the rate of phosphorylation is proportional to both the kinase concentration and the concentration of free phosphorylation sites nP-r. The solution is given by $r = nP\left(1 - exp\left(-\beta Pt\right)\right)$. Since a fraction f of the initial reference concentration was loaded for quantification, the measured concentrations were first multiplied by $1/f$ and then fitted for the function $aP(1\text{-}e^{-bP})$ with a and b as fitting parameters. Testing for cis-phosphorylation was done by replacing the term βP with βP+ω, where ω is the cis-phosphorylation rate.

We do not know if and how β depends on the phosphorylation state of Pom1. The choice to have β independent from the number of available sites was mainly motivated by the wish to keep the model mathematically tractable. However, this assumption is unlikely to have a large effect on the model, since it impacts mostly the phosphorylation rates of highly phosphorylated Pom1, which anyway makes up a small fraction of cortical Pom1 as it detaches fast from the membrane. Technically speaking, note that β usually appears in conjunction with κi in the equations, so that we could potentially allow for varying βi but then make assumptions on κi/βi instead of κi.

MODEL (Supplementary Text S1):

*Figure 1 is missing the detachment of rate for P0, which goes back to the cytoplasm with k0*P0.*

This is intended, as we assume that k0 is zero. It has indeed been shown that non-phosphorylated Pom1 does not form a gradient and binds lipids at the plasma membrane with much greater affinity than phosphorylated Pom1. These data suggest that its detachment is negligible and can be ignored (Hachet et al, 2009). We clarified this in the text and in the equations.

The overall scheme of chemical reactions is a-priori not thermodynamically consistent. If the release of Pom1 from the cortex occurs passively as described (i.e. from changes in electrostatic interactions), then one would also expects cytoplasmic unbound phosphorylated Pom1 to be able to rebind directly to the cortex. Thus for every species there should be a equilibrium, with two reverse reactions. It may be of course possible to deduce that the binding fluxes are small, of to argue in this direction in some way.

The mechanisms underlying Pom1 detachment from the membrane have not yet been elucidated, so it could be passive or active. We can of course not exclude that there is reattachment of unbound, phosphorylated Pom1 to the membrane. However, this must be minimal because in *tea4* mutant cells, in which Pom1 cannot be dephosphorylated, Pom1 remains almost entirely cytoplasmic (Hachet et al, 2009; Tatebe et al, 2005). We can thus safely neglect this.

In equation (4) it seems that the sum for i should start at one and not zero, or S should appear in the equation. If was not clear how equation (4) is used in the rest of the analysis.

We thank the reviewer spotting this and we changed the sum to start from 1. Since we assume that k0 is zero, it would not make a difference if the sum started from zero, but we agree that it is better not to use this fact in writing out the sum. Note that this equation is used to justify our estimate of gamma. We expanded this more rigorously in the present version.

*In "1. Gradient shape", applying boundary conditions is not valid here. Instead, one may use conservation of mass (the number of pom1p) to set the amplitude coefficients. The assumption of an infinite cell length should hold, but it can be justified more rigorously (L*lambda greater than 1).*

We realize that our wording was misleading and changed it accordingly. *A* is indeed not a boundary condition that we imposed, rather we define it as the gradient amplitude at x=0, without further consequence.

In "2. Source quantification". The assumption that the number of Pom1 molecules attaching to the membrane is proportional to the Tea4 concentration violates basic chemical kinetic theory. This rate should also depend on the concentration of Pom1 in the cytoplasm. Conservation of Pom1 imply that the cytoplasmic concentration is inversely correlated with the total amount of Pom1 on the cortex. The cell is a closed system and this automatically provides a certain amount of 'buffering'. This important point concerns all the results of the paper.

The rate of Pom1 dephosphorylation (and thus attachment to the cortex) must indeed depend on both the concentrations of the phosphatase (the Tea4-Dis2 complex) and the substrate (phosphorylated, cytosolic Pom1), at least if this reaction follows simple Michaelis-Menten kinetics. Enzymatic reactions also display saturation kinetics, such that the reaction reaches a maximal plateau at high substrate concentrations. As described in Hachet et al, 2009, cytoplasmic Pom1 binds to and is dephosphorylated by the Tea4-Dis2 complex, which was actively brought to the cell pole by the microtubules. It is thus reasonable to assume that the concentration of Tea4 at the pole is a good proxy for the amount of Pom1 that is deposited at the pole. Indeed, the strong correlation between Tea4 and Pom1 amplitudes supports this assumption. The influence of the cytosolic Pom1 concentration on Pom1 attachment at the cell pole is less clear. In fact, we observe no correlation between the amplitude of Pom1 at the pole and Pom1 concentration in the cytoplasm, a point particularly well illustrated in cells displaying very important amplitude differences at the two cell poles that share the same cytosol (see Fig S1). One likely explanation is that cytosolic Pom1 is at saturating levels for the de-phosphorylation reaction, such that the reaction runs at maximal enzymatic rate and is only (or primarily) dependent on enzyme concentration. We do not know at what concentration Pom1 becomes saturating, but we noted above that Pom1 cytosolic concentration is far from negligible. Additionally, the demonstrated direct binding of Pom1 to the phosphatase regulatory subunit Tea4 may contribute to increasing the local Pom1 concentration in the immediate vicinity of the enzyme. These observations and considerations suggest that the amount of Pom1 that is brought to the pole depends mainly on the (largely stochastic) microtubule dynamics, which deliver Pom1 by bursts.

Overall, the Supplementary Text S1 should be clarified by adding a justification for each part. In its present form, it looks like an exercise in algebra. One should state the assumptions, declare the result to be proven, before proceeding with the proof. Finally, it is important to first discuss a model based on the laws of chemistry, even if one can justify assumptions to depart from it.

We did our best to clarify the context and the assumptions of our model. We added an introductory paragraph in each section to clearly state what is to be achieved in it. Consistent with a large body of literature on gradient formation in biological systems, we adopt a formalism and a presentation style that is more in line with the tradition of physics than biochemistry. This of course does not hinder us from giving due considerations to the laws of chemistry.

Reviewer #2:

This manuscript examines how the protein kinase Pom1 forms a spatial gradient at the plasma membrane of fission yeast cells. These authors previously showed that Pom1 autophosphorylation is a key step in gradient formation because it controls Pom1-membrane interactions (Hachet et al., Cell, 2011). This mechanism was included in a subsequent mathematical model for gradient assembly published by others (Saunders et al., 2012). A key step in this previous model was formation of dynamic clusters, which were proposed to allow robust gradient assembly in the presence of Pom1 concentration fluctuations. In this current manuscript, the authors add one new conceptual component to a model for Pom1 gradient assembly. Specifically, autophosphorylation can occur through intramolecular/cis phosphorylation, or alternatively through intermolecular/trans phosphorylation. The distinction is important because intramolecular autophoshorylation should be concentration independent, while the rate/extent of intermolecular autophosphorylation will depend on protein concentration. This has major implications in a system like the Pom1 gradient, where the protein diffuses away from a point source as a dephosphorylated species. In my view, this is an extremely important concept that should be considered key to how this gradient forms, with relevance to how gradients form in other biological systems as well. The paper contains a combination of cell biology, biochemistry, and mathematical modeling to make this point. I have only minor comments that might be considered to strengthen the manuscript.

1. The in vitro case for trans-autophosphorylation (as opposed to cis) could be strengthened. The concentration dependence shown in Figure 3A is consistent with trans phosphorylation, with additional contributions from cis as well. However, the authors could clearly show trans phosphorylation in vitro using purified Pom1 and GST-Pom1(kinase-dead). In the trans model, active wildtype protein will phosphorylate the purified kinase-dead protein. Adding something like a GST tag should move the kinase-dead protein to a region of the gel that separates it from the wildtype protein.

We have performed the suggested experiment using recombinant Pom1 and recombinant kinase-dead truncated Pom1 lacking the first 305 amino acids (which we showed earlier is largely functional in vivo: Bhatia et al, 2014). This experiment indeed shows that Pom1 can phosphorylate the inactive Pom1 allele, thus confirming inter-molecular phosphorylation.

2. The data support a role for trans autophosphorylation but do not exclude a role for cis autophosphorylation. The authors should clearly state in the text that their model does not exclude the presence or potential role of cis-phosphorylation in the system. Since trans versus auto phosphorylation

should compete for the same sites on Pom1, I also wonder if the authors can use their model to estimate the amount of trans versus cis in cells.

We agree that we cannot exclude that Pom1 auto-phosphorylates in *cis*. We clarified this in the discussion stating that

> "This, however, does not exclude that Pom1 also auto-phosphorylates intra-molecularly."

Regarding the proportion of trans vs cis phosphorylation, the quality of the prediction indicate that cis-phosphorylation can be neglected at the Pom1 concentration encountered close to the cell poles. At lower concentrations, close to the cell middle it could be more important but the model alone cannot be used to assess this. As mentioned above, the *in vitro* data is also not univocal about this.

2nd Editorial Decision 18 May 2015

Thank you again for submitting your revised work to Molecular Systems Biology. We have now finally heard back from the two referees who agreed to evaluate your revision. While reviewer #2 is now satisfied, reviewer #1 still raises points with regard to the presentation of the data.

There are 2 main issues remaining:

- first, pom1 levels are described as "highly" variable in the paper whereas referee #1 disagrees with this qualification. While the issue could be seen as merely semantic, it seems important to us to clarify more objectively the degree of Pom1 variability with regard to the expected variability due to cell size effects.

- the second issue relates to the assumption that the decay profiles fit an exponential decay (ie in the calculation of decay length), in which case total P should be proportional to L*A. This seems to be inconsistent with the data shown in Fig 1E, and Fig S1, as explained by reviewer #1 below.

If you feel you can satisfactorily deal with these points and those listed by the referees, you may wish to submit a revised version of your manuscript. Please attach a covering letter giving details of the way in which you have handled each of the points raised by the referees. A revised manuscript might be once again subject to review and you probably understand that we can give you no guarantee at this stage that the eventual outcome will be favorable.

Reviewer #1:

The authors have not followed my main suggestion but nevertheless improved their manuscript. The supplementary document describing the model is now very clear and this is a huge improvement. I have read the response, and agree that the rate at which Pom1 is loaded could be independent of Pom1 cytoplasmic concentration in some regime, in particular if the limiting factors are for example deposited by microtubules. So this partly relieves my previous criticism.

I disagree however with the statements that "pom1 levels are highly variable between cells": Fig 1C indicate that 90% of the cells fall within a fold-change that is about 2: once the two peak curves are removed, which leaves 90% of the data, all peak values are between ~1200 and ~2800.

I also disagree that "pom1 levels at cell poles are highly variable within the same cell". Fig S1B indicates that only 5 cells in the dataset have a ratio bigger than 4. It seems that in 50% of all cells, the two gradients have amplitudes that only differ at most by a factor 2.

The variability in total Pom1 content across all cells (Fig S1A) is also about a factor 2 (std-dev / mean). On the X-axis of Fig.S1C & D we can read a total variation for the cytoplasmic concentration of Pom1 of ~0.4 log-units, corresponding to 50% variation (that is less than a factor 2).

As the authors rightly point-out, a factor 2 of variation in the total is the minimum that we should expect, if concentration was constant, considering that cells double in volume during their cycle, and that cells of all lengths were used for this study. Arguably, this is a qualitative statement, but overall, this indicates IMHO a rather precise control of the Pom1p concentration across the population (for different cells).

So I am not satisfied by the answer provided in the response to my comments: "In this context, an inversely proportional relationship between A and lambda on the basis of a constant P would have to assume that P does not change across cells. We examined whether this assumption is supported by our data and it appears not to be the case."

Moreover, I was utterly confused by some of the data presented, and after spending a significant amount of time trying to make sense of it, I have to give up. I explain below what puzzles me, with a few suggestions that may be useful to improve the manuscript:

The gradient is defined by an "amplitude at the pole" A, and a "decay length" L.
Generally, we expect that the total protein count in the gradient would be proportional to LA.
It should correspond to what the authors call "Total pom1 in gradient"
Let's call P the cytoplasmic Pom1 concentration.

Fig. 1E shows an anti-correlation: L ~ A ^ (-1/2)
Fig. S1C shows a correlation: LA ~ P ^ 0.6
Fig. S1D shows no correlation: A ~ P ^ 0
Fig. S1F shows a strong correlation: A ~ (LA)^1.5

My confusion is that all these relations are not compatible, and yet it seems that the different plots present the same data in different ways.
For example, by substituting the relationship from Fig. 1E into that of S1F, we can derive:
A ~ (L A) ^ 1.5
A ~ ((A ^ -0.5) * A) ^ 1.5
A ~ (A ^ 0.5) ^ 1.5
A ~ A ^ 0.75
Which is problematic.

Similarly, multiplying the relation from Fig. 1E by L, one can derive:
LA ~ A ^ 0.5
and now using the relationship from Fig. S1C on the left-hand side
P ^ 0.6 ~ A ^ 0.5
hence
A ~ P ^ 1.2
which is in stark contradiction with Fig. S1D

I understand that the "Total cortical Pom1" is the sum of the "Total pom1 in gradient" from the two gradients in one cell, but I do not see how this could change the overall scaling behavior. If this would be the case, it would be wise to plot the "Total pom1 in gradient" on Fig. S1C.
I also see that Figure 1E and S1F are based on the averaged profiles, while Fig. S1C and D are based on non-averaged data points, but I see no reason for this to change the scaling relation between the measured quantities. It would be actually very worrying if the averaged quantities did not exhibit the same scaling as the non-averaged ones. It would be useful to make a plot similar to Fig S1D, but summing the two amplitudes in each cell for the Y-axis.

The "Total pom1 in gradient" should correspond to L*A, for an exponential profile in 1D, and the profiles are said to be well fitted by exponentials (Fig. S6). The geometry of the cell affects the total amount of Pom1 in the gradient, since the high value correspond to the curved tip of the cell which has a reduced cortical area. The formula used by the author (sum{p_i*r_i}) is correct, but I am unable to assess what different this makes on the total, compared to simply using L*A. To address this point, one could make a scatter plot, with L*A on one axis, and the "Total pom1 in gradient" on the other.

In conclusion, I am very confused, and it seems that some fundamental inconsistency remains. Importantly, this criticism does not relate to the model, which is now quite convincing, but rather to the data analysis or to its presentation. I am sorry not to have been able to spot this during my initial review, but the data that is now presented in Figure S1 was not available then. This data is very interesting and valuable.

Further suggestions:

I would suggest to use a scatter plot instead of the histogram for Fig S1B.

The axis of Fig S1A should be explained: Saunders et al. seem to report 5000 pom1p molecules per cell on average. Marguerat S et al. (2012) report 1500 copies/cell. What is the "Total Pom1" which

has a value of 7e+07?

Fig. S1C, D, F are log-log plot, and it would be better to use equal axis magnifications, so that slopes become apparent.

Please, provide a regression on Fig S1D.

Reviewer #2:

I am satisfied with the authors' responses to my and the other reviewer's concerns. At this stage, I support publication of this nice work.

Revision received April 30 2015

Response to Reviewer #1:

The authors have not followed my main suggestion but nevertheless improved their manuscript. The supplementary document describing the model is now very clear and this is a huge improvement. I have read the response, and agree that the rate at which Pom1 is loaded could be independent of Pom1 cytoplasmic concentration in some regime, in particular if the limiting factors are for example deposited by microtubules. So this partly relieves my previous criticism.

We are happy we were able to clarify this aspect of our work and thank the reviewer for asking us to do so.

I disagree however with the statements that "pom1 levels are highly variable between cells": Fig 1C indicate that 90% of the cells fall within a fold-change that is about 2: once the two peak curves are removed, which leaves 90% of the data, all peak values are between ~1200 and ~2800.

I also disagree that "pom1 levels at cell poles are highly variable within the same cell". Fig S1B indicates that only 5 cells in the dataset have a ratio bigger than 4. It seems that in 50% of all cells, the two gradients have amplitudes that only differ at most by a factor 2.

The variability in total Pom1 content across all cells (Fig S1A) is also about a factor 2 (std-dev / mean). On the X-axis of Fig.S1C & D we can read a total variation for the cytoplasmic concentration of Pom1 of ~0.4 log-units, corresponding to 50% variation (that is less than a factor 2).

Throughout the paper, the displayed logarithms are in base 10 (not the natural logarithm) such that a 0.4 range corresponds to a 2.5 fold change. We apologize for having omitted to mention this and corrected the figures legends accordingly. We also reformulated the text to be more objective and precise regarding the variability of Pom1 in the cells:

"Across cells, we observe a variation of cortical and total Pom1 amounts, as well as cytoplasmic Pom1 concentration slightly above two-fold, as would be expected from cells that repeatedly half and then double their volume along the cell cycle, hinting at a possible control of Pom1 production and degradation. Pom1 levels at cell poles display a somewhat higher variability with up to several fold differences in amplitude across cells (Saunders et al, 2012) (Fig. S1D), and up to four-fold differences within cells (Fig S1B)."

As the authors rightly point-out, a factor 2 of variation in the total is the minimum that we should expect, if concentration was constant, considering that cells double in volume during their cycle, and that cells of all lengths were used for this study. Arguably, this is a qualitative statement, but overall, this indicates IMHO a rather precise control of the Pom1p concentration across the population (for different cells).

So I am not satisfied by the answer provided in the response to my comments: "In this context, an inversely proportional relationship between A and lambda on the basis of a constant P would have to assume that P does not change across cells. We examined whether this assumption is supported by our data and it appears not to be the case."

© European Molecular Biology Organization

We agree that the overall Pom1 amount could be controlled and now mention this hypothesis explicitly in the text (see excerpt above). However, such a control would not keep the amount of cortical Pom1 constant across cells. It would at best maintain it within a two-fold range. Since such a range is of comparable magnitude to the variability of the decay length, this control cannot be invoked to explain the anti-correlation between gradient amplitude at the tip and the decay length.

Moreover, I was utterly confused by some of the data presented, and after spending a significant amount of time trying to make sense of it, I have to give up. I explain below what puzzles me, with a few suggestions that may be useful to improve the manuscript:

The gradient is defined by an "amplitude at the pole" A, and a "decay length" L. Generally, we expect that the total protein count in the gradient would be proportional to LA. It should correspond to what the authors call "Total pom1 in gradient"

Let's call P the cytoplasmic Pom1 concentration.

Fig. 1E shows an anti-correlation: $L \sim A^{(-1/2)}$

Fig. S1C shows a correlation: $LA \sim P^{0.6}$

Fig. S1D shows no correlation: $A \sim P^{0}$

Fig. S1F shows a strong correlation: $A \sim (LA)^{1.5}$

My confusion is that all these relations are not compatible, and yet it seems that the different plots present the same data in different ways.

For example, by substituting the relationship from Fig. 1E into that of S1F, we can derive:

$A \sim (LA)^{1.5}$

$A \sim ((A^{-0.5}) * A)^{1.5}$

$A \sim (A^{0.5})^{1.5}$

$A \sim A^{0.75}$

Which is problematic.

This is indeed a valid point and we thank the reviewer for raising it. We redid the computation for the "total Pom1 in gradient" and found out that it is actually proportional to $A^{0.5}$ (see section 5 in our revised supplementary text). So we have $A \sim (LA)^2$ and the 1.5 in the above derivation should be replaced by a 2. This solves the issue raised by the reviewer, but raises the question why our data indicates that $A \sim (LA)^{1.5}$. We explain this as follow. Theoretically LA is the total area under a gradient of infinite length, but our averaged gradients are cut at 3.2 microns as shown in Fig. 1C. So it is not really the total area under the gradient that we were presenting in Fig. S1F but rather the "area under the gradient up to 3.2 microns". We performed simulations to test our hypothesis that the truncation of gradients beyond a position x could explain the smaller observed exponent in the power-law describing the relationship between the area under the curve and the amplitude of the profile (see graph below). As expected, the value for the exponent is trivially one when x tends to zero and two when x becomes very large. In our data, λ is on average is around 1.4 microns (see Fig. 1E) so we have $x/\lambda = 3.2/1.4 = 2.3$, which according to the simulations should lead to a 1.57 power law (blue line in graph below), consistent with our observations at 1.52. Since this is not the most intuitive way to present our data, we decided to directly regress (in log space) the total cortical Pom1 against the mean amplitude at the pole (see new Fig. S1F). This yields a slope of 0.46, in accordance with the model prediction of 0.5.

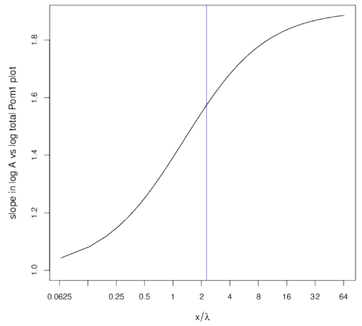

Similarly, multiplying the relation from Fig. 1E by A, one can derive:

$LA \sim A^{0.5}$

and now using the relationship from Fig. S1C on the left-hand side

$P^{0.6} \sim A^{0.5}$

hence

$A \sim P^{1.2}$

which is in stark contradiction with Fig. S1D

The above line of reasoning is only correct if the proportionality factor in the top equation (from Fig. 1E) is assumed to be independent from P. Clearly, the modeling of how exactly cytoplasmic Pom1 is recruited to the cell poles is beyond the scope of this paper, so our model does not say anything regarding the relationship between the cytoplasmic concentration P and the amplitude of Pom1 at the pole or the cortical Pom1. We also point out that slope of the regression line in Fig. S1C is 0.53, which we approximate as 0.5.
Since P does not appear in the model, we can make no assumption on its effect or lack thereof on the cortical Pom1. Moreover, while Fig. S1C shows a correlation between P and the total cortical Pom1, it does not exclude other additional effects on the total cortical Pom1. For example, we could have
$\log \mathrm{LA} = 0.5 * \log \mathrm{A} + 0.5 * \log \mathrm{P}$, *which is equivalent to* $\mathrm{LA} \sim \mathrm{A}^{0.5}\mathrm{P}^{0.5}$.
This relationship is compatible with all our data, including Fig. 1E, Fig. S1C and Fig. S1D, although this is not a claim we want to make in the paper. Indeed, when simultaneously regressing (in log space) the total cortical Pom1 against both the mean amplitude at the poles and the cytoplasmic Pom1 concentration, we find slopes 0.41 and 0.45 with very significant p-values and a total R^2 of 60%. But it may also well be that A and P are indeed slightly correlated but that we lack the power of show it.
I understand that the "Total cortical Pom1" is the sum of the "Total pom1 in gradient" from the two gradients in one cell, but I do not see how this could change the overall scaling behavior. If this would be the case, it would be wise to plot the "Total pom1 in gradient" on Fig. S1C.
I also see that Figure 1E and S1F are based on the averaged profiles, while Fig. S1C and D are based on non-averaged data points, but I see no reason for this to change the scaling relation between the measured quantities. It would be actually very worrying if the averaged quantities did not exhibit the same scaling as the non-averaged ones.
Our new Fig. S1F shows that the non-averaged data is also consistent with the model.
It would be useful to make a plot similar to Fig S1D, but summing the two amplitudes in each cell for the Y-axis.
As expected, the plot of the sum of the two amplitudes versus cytoplasmic Pom1 concentration is very similar to Fig. S1D.

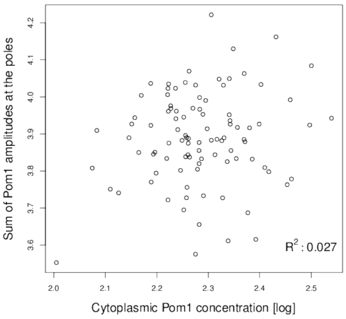

*The "Total pom1 in gradient" should correspond to L*A, for an exponential profile in 1D, and the profiles are said to be well fitted by exponentials (Fig. S6). The geometry of the cell affects the total amount of Pom1 in the gradient, since the high value correspond to the curved tip of the cell which has a reduced cortical area. The formula used by the author (sum{p_i*r_i}) is correct, but I am unable to assess what different this makes on the total, compared to simply using L*A. To address this point, one could make a scatter plot, with L*A on one axis, and the "Total pom1 in gradient" on the other.*
In conclusion, I am very confused, and it seems that some fundamental inconsistency remains. Importantly, this criticism does not relate to the model, which is now quite convincing, but rather to the data analysis or to its presentation. I am sorry not to have been able to spot this during my initial review, but the data that is now presented in Figure S1 was not available then. This data is very interesting and valuable.
We thank the reviewer for uncovering an inconsistency in the presentation of our data and thus give us the chance to improve this paper. We hope our explanations and corrections help clarifying all issues raised.

Further suggestions:
I would suggest to use a scatter plot instead of the histogram for Fig S1B.
We changed the figure accordingly.

The axis of Fig S1A should be explained: Saunders et al. seem to report 5000 pom1p molecules per cell on average. Marguerat S et al. (2012) report 1500 copies/cell. What is the "Total Pom1" which has a value of 7e+07?
The reported numbers correspond to signal intensities which are assumed to be proportional to the number of molecules. Since we do not wish to make a claim on the actual number of molecules in the cell, we specified that those units are arbitrary.
Fig. S1C, D, F are log-log plot, and it would be better to use equal axis magnifications, so that slopes become apparent. Please, provide a regression on Fig S1D.
We changed the figure accordingly. The regression line for Fig S1D is not significantly different from zero and we find it misleading to draw such a non significant slope. We therefore only indicate the R2 value. The panel with the regression line and confidence interval is provided below and can be inserted in Fig S1D if required.

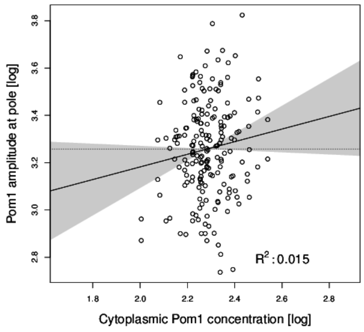

Acceptance letter 04 June 2015

Thank you again for sending us your revised manuscript. We are now satisfied with the modifications made and I am pleased to inform you that your paper has been accepted for publication. Thank you very much for submitting your work to Molecular Systems Biology.

Reviewer #1:

The article was improved, and indeed it was important to recognise the potential effects due to the cytoplasmic Pom1 pool. Frankly, I do not agree with the argument that total Pom1 amount in the gradient (LA) may have a dependency on the cytoplasmic pool of Pom1 (P) that is not already captured in the maximum amplitude (A). The observation that LA scales like A^0.5 * P^05, is problematic considering the scenario of gradient formation offered in the article. If Pom1 is indeed loaded at the tip of the cell, and only there, then the dependency of A on P should completely determine how the gradient depends on P. The observed scaling relations thus likely indicate that some aspects of the systems are still not understood, and they are certainly not included in the model. As always, more work is needed! The submitted article is nevertheless a very valuable contribution, and as I said in my original review should be interesting to a wide audience. I apologise for the delay in my re-evaluation. If I was permitted to be picky, I would ask for log-log plots to be presented always with equal axis (Figure S1), but if the underlying data is made available to readers, this is maybe not essential. I wish to congratulate the authors on the nice story.
